# Supplementary material for: Intestinal inflammation induced by heat-labile toxin-producing enterotoxigenic E: Coli infection and impact on immune responses in an experimental human challenge model
Source: PLoS Negl Trop Dis. 2025 Oct 3;19(10):e0013025. doi: 10.1371/journal.pntd.0013025 (PMC12510637; doi:10.1371/journal.pntd.0013025)
Supplement: S1 Fig — (DOCX) [file pntd.0013025.s001.docx]

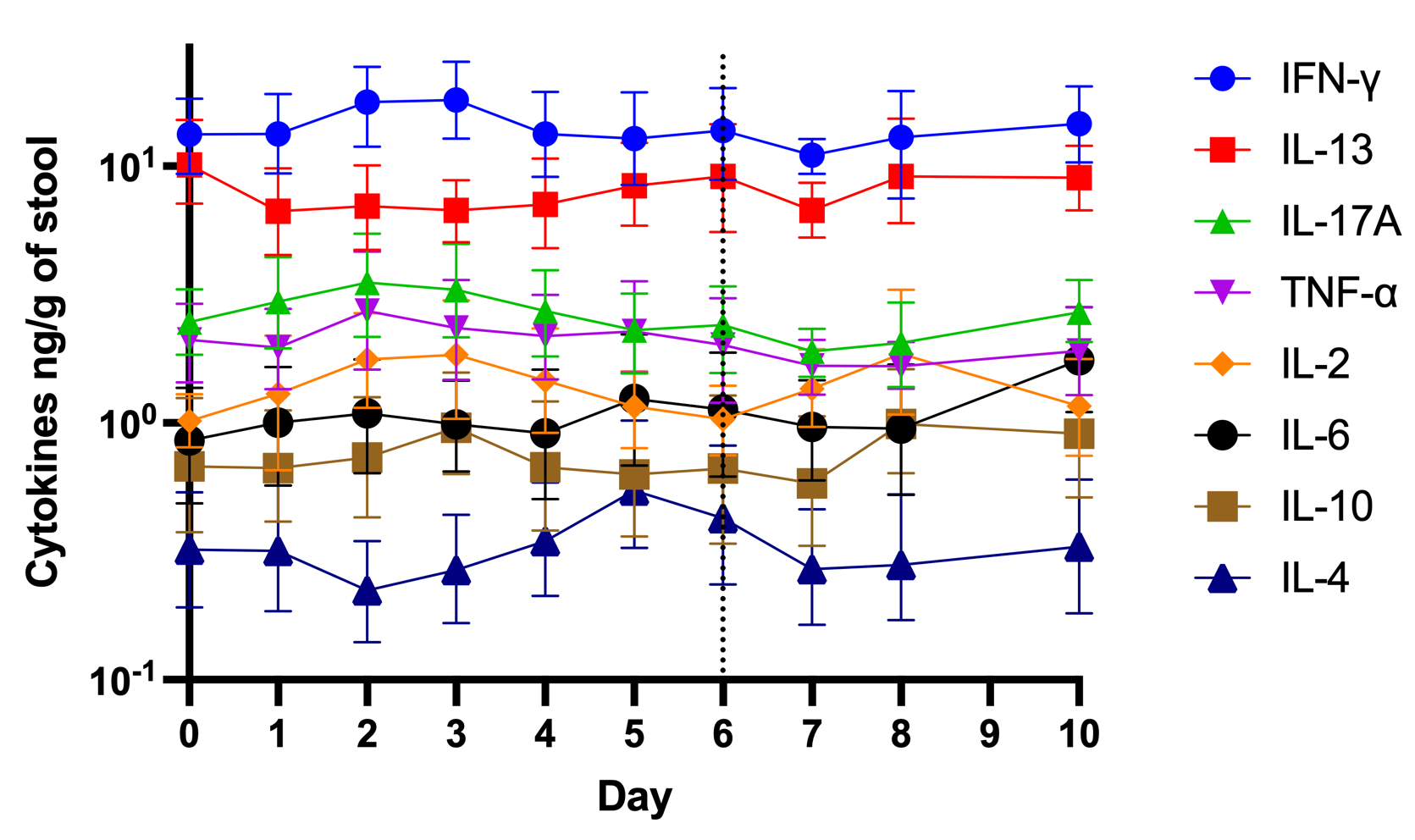


**Supplemental Fig 1.** Levels of fecal cytokine IFN-γ, IL-13, IL-17A, TNF-α, IL-2, IL-6, IL-10, and IL-4 pre- and post ETEC challenge among all participants (log 10 scale). D0: day before challenge; 1 to 9: 1 to 9 days after challenge. Note, the dashed vertical line at Day 6 denotes when all participants were receiving antibiotic treatment.

Fecal cytokine titers peaked at 4.42-fold for IL-2 (p=0.003), 2.41-fold for IL-4 (p=0.003), 3.19-fold for IL-6 (p=0.003), 2.43-fold for IL-10 (p=0.004), 1.40-fold for IL-13 (p=0.036), 2.71-fold for IL-17A (p=0.004), 2.17-fold for IFN-γ (p=0.006), and 2.73-fold for TNF-α (p=0.004) compared to baseline. A significant increase was also seen among MSDs in cytokine IL-2 (p=0.014), IL-4 (p=0.014), IL-6 (p=0.014), IL-10 (p=0.014), IL-17A (p=0.022), IFN-γ (p=0.022) and TNF-α (p=0.014) when comparing peak values and baseline values.
