# Supplementary material for: Intestinal inflammation induced by heat-labile toxin-producing enterotoxigenic E: Coli infection and impact on immune responses in an experimental human challenge model
Source: PLoS Negl Trop Dis. 2025 Oct 3;19(10):e0013025. doi: 10.1371/journal.pntd.0013025 (PMC12510637; doi:10.1371/journal.pntd.0013025)
Supplement: S1 Table — (DOCX) [file pntd.0013025.s002.docx]

**Supplemental Table 1**. Correlation between MPO and cytokines

| **MPO** | **Cytokines** | **Correlation Estimate** | **P Value** |
| --- | --- | --- | --- |
| Peak MPO | Baseline IL-1β | -0.099 | 0.736 |
|  | Peak IL-1β | 0.653 | 0.011 |
|  | Peak IL-1β fold change | 0.402 | 0.154 |
|  | Baseline IL-2 | 0.072 | 0.807 |
|  | Peak IL-2 | 0.143 | 0.626 |
|  | Peak IL-2 fold change | 0.185 | 0.527 |
|  | Baseline IL-4 | 0.091 | 0.757 |
|  | Peak IL-4 | -0.301 | 0.296 |
|  | Peak IL-4 fold change | -0.394 | 0.164 |
|  | Baseline IL-6 | 0.097 | 0.742 |
|  | Peak IL-6 | -0.077 | 0.794 |
|  | Peak IL-6 fold change | 0.092 | 0.753 |
|  | Baseline IL-8 | -0.037 | 0.899 |
|  | Peak IL-8 | 0.675 | 0.008 |
|  | Peak IL-8 fold change | 0.477 | 0.085 |
|  | Baselien IL-10 | -0.060 | 0.840 |
|  | Peak IL-10 | 0.266 | 0.358 |
|  | Peak IL-10 fold change | 0.417 | 0.138 |
|  | Baseline IL-13 | 0.157 | 0.593 |
|  | Peak IL-13 | 0.244 | 0.401 |
|  | Peak IL-13 fold change | 0.044 | 0.882 |
|  | Baseline IL-17A | 0.331 | 0.248 |
|  | Peak IL-17A | 0.205 | 0.483 |
|  | Peak IL-17A fold change | 0.148 | 0.614 |
|  | Baseline TNF-α | -0.326 | 0.255 |
|  | Peak TNF-α | 0.143 | 0.626 |
|  | Peak TNF-α fold change | 0.461 | 0.097 |
|  | Baseline IFN-γ | -0.279 | 0.334 |
|  | Peak IFN-γ | 0.051 | 0.864 |
|  | Peak IFN-γ fold change | 0.380 | 0.180 |
| Peak MPO fold change | Baseline IL-1β | 0.033 | 0.911 |
|  | Peak IL-1β | 0.657 | 0.011 |
|  | Peak IL-1β fold change | 0.292 | 0.311 |
|  | Baseline IL-2 | 0.040 | 0.891 |
|  | Peak IL-2 | 0.191 | 0.513 |
|  | Peak IL-2 fold change | 0.255 | 0.379 |
|  | Baseline IL-4 | -0.156 | 0.595 |
|  | Peak IL-4 | -0.420 | 0.135 |
|  | Peak IL-4 fold change | -0.257 | 0.374 |
|  | Baseline IL-6 | -0.135 | 0.646 |
|  | Peak IL-6 | -0.240 | 0.409 |
|  | Peak IL-6 fold change | 0.128 | 0.664 |
|  | Baseline IL-8 | 0.033 | 0.911 |
|  | Peak IL-8 | 0.653 | 0.011 |
|  | Peak IL-8 fold change | 0.429 | 0.126 |
|  | Baselien IL-10 | -0.285 | 0.324 |
|  | Peak IL-10 | 0.103 | 0.725 |
|  | Peak IL-10 fold change | 0.501 | 0.068 |
|  | Baseline IL-13 | 0.002 | 0.994 |
|  | Peak IL-13 | 0.051 | 0.864 |
|  | Peak IL-13 fold change | 0.005 | 0.987 |
|  | Baseline IL-17A | 0.302 | 0.294 |
|  | Peak IL-17A | 0.095 | 0.748 |
|  | Peak IL-17A fold change | 0.020 | 0.946 |
|  | Baseline TNF-α | -0.512 | 0.061 |
|  | Peak TNF-α | 0.051 | 0.864 |
|  | Peak TNF-α fold change | 0.501 | 0.068 |
|  | Baseline IFN-γ | -0.384 | 0.175 |
|  | Peak IFN-γ | -0.125 | 0.669 |
|  | Peak IFN-γ fold change | 0.331 | 0.247 |
| Baseline MPO | Baseline IL-1β | -0.411 | 0.144 |
|  | Peak IL-1β | -0.323 | 0.260 |
|  | Peak IL-1β fold change | 0.073 | 0.805 |
|  | Baseline IL-2 | -0.327 | 0.253 |
|  | Peak IL-2 | -0.116 | 0.692 |
|  | Peak IL-2 fold change | 0.059 | 0.840 |
|  | Baseline IL-4 | 0.527 | 0.053 |
|  | Peak IL-4 | 0.147 | 0.615 |
|  | Peak IL-4 fold change | -0.271 | 0.349 |
|  | Baseline IL-6 | 0.490 | 0.075 |
|  | Peak IL-6 | 0.477 | 0.085 |
|  | Peak IL-6 fold change | -0.117 | 0.691 |
|  | Baseline IL-8 | 0.086 | 0.771 |
|  | Peak IL-8 | -0.226 | 0.436 |
|  | Peak IL-8 fold change | -0.182 | 0.533 |
|  | Baselien IL-10 | 0.483 | 0.080 |
|  | Peak IL-10 | 0.152 | 0.605 |
|  | Peak IL-10 fold change | -0.201 | 0.491 |
|  | Baseline IL-13 | 0.408 | 0.147 |
|  | Peak IL-13 | 0.253 | 0.383 |
|  | Peak IL-13 fold change | -0.134 | 0.648 |
|  | Baseline IL-17A | -0.211 | 0.469 |
|  | Peak IL-17A | -0.108 | 0.714 |
|  | Peak IL-17A fold change | -0.011 | 0.970 |
|  | Baseline TNF-α | 0.321 | 0.262 |
|  | Peak TNF-α | 0.020 | 0.946 |
|  | Peak TNF-α fold change | -0.192 | 0.511 |
|  | Baseline IFN-γ | 0.368 | 0.195 |
|  | Peak IFN-γ | 0.033 | 0.911 |
|  | Peak IFN-γ fold change | -0.191 | 0.513 |
