# Supplementary material for: Intestinal inflammation induced by heat-labile toxin-producing enterotoxigenic E: Coli infection and impact on immune responses in an experimental human challenge model
Source: PLoS Negl Trop Dis. 2025 Oct 3;19(10):e0013025. doi: 10.1371/journal.pntd.0013025 (PMC12510637; doi:10.1371/journal.pntd.0013025)
Supplement: S2 Table — (DOCX) [file pntd.0013025.s003.docx]

**Supplemental Table 2**. Number and percentage of immune responders

|  | IgA | IgG |
| --- | --- | --- |
| CS17 serum | 12 (80%) | 13 (87%) |
| CTB serum | 6 (40%) | 6 (40%) |
| CS17 ALS | 15 (100%) | ND |
| CS17 fecal | 10 (67%) | ND |
| CTB ALS | 9 (60%) | ND |
| CTB fecal | 11 (73%) | ND |

ND: not done
