# Supplementary material for: Intestinal inflammation induced by heat-labile toxin-producing enterotoxigenic E: Coli infection and impact on immune responses in an experimental human challenge model
Source: PLoS Negl Trop Dis. 2025 Oct 3;19(10):e0013025. doi: 10.1371/journal.pntd.0013025 (PMC12510637; doi:10.1371/journal.pntd.0013025)
Supplement: S3 Table — (DOCX) [file pntd.0013025.s004.docx]

**Supplemental Table 3**. MPO levels by seroconversion status

|  |  |  | **Seroconversion status** | |  |
| --- | --- | --- | --- | --- | --- |
| **MPO** | **Antibody** | **Antibody fold change cutoff** | **Non-responder** | **Responder** | **P Value** |
| Baseline MPO | CS17 serum IgA | 2 | 436.5 (254.5-1016.2) | 318.2 (108.5-647.5) | 0.734 |
| Baseline MPO | CS17 serum IgA | 4 | 336.7 (225.5-1016.2) | 340.5 (108.5-647.5) | 0.388 |
| Baseline MPO | CS17 serum IgA | 8 | 304.4 (108.5-1016.2) | 420.4 (262-647.5) | 0.254 |
| Baseline MPO | CS17 serum IgG | 4 | 282.6 (108.5-647.5) | 382.6 (254.5-1016.2) | 0.529 |
| Baseline MPO | CS17 serum IgG | 8 | 348.7 (108.5-1016.2) | 320.3 (254.5-521.5) | 0.679 |
| Baseline MPO | CS17 ALS IgA | 16 | 385 (254.5-1016.2) | 323.6 (108.5-647.5) | 1 |
| Baseline MPO | CS17 ALS IgA | 32 | 345.9 (225.5-1016.2) | 335.5 (108.5-647.5) | 0.513 |
| Baseline MPO | CS17 ALS IgA | 64 | 370.4 (225.5-1016.2) | 319.5 (108.5-647.5) | 0.955 |
| Baseline MPO | CS17 fecal IgA | 8 | 442 (264.2-1016.2) | 317.1 (108.5-647.5) | 0.6 |
| Baseline MPO | CS17 fecal IgA | 16 | 451.2 (264.2-1016.2) | 269.8 (108.5-521.5) | 0.23 |
| Baseline MPO | CS17 fecal IgA | 32 | 441.1 (264.2-1016.2) | 225.8 (108.5-311.2) | 0.017 |
| Baseline MPO | CTB serum IgA | 2 | 362.6 (254.5-647.5) | 306.3 (108.5-1016.2) | 0.456 |
| Baseline MPO | CTB serum IgG | 2 | 304.6 (108.5-647.5) | 397.9 (225.5-1016.2) | 0.607 |
| Baseline MPO | CTB ALS IgA | 4 | 375.4 (264.2-647.5) | 316.6 (108.5-1016.2) | 0.272 |
| Baseline MPO | CTB ALS IgA | 8 | 342.4 (254.5-647.5) | 333.8 (108.5-1016.2) | 0.864 |
| Baseline MPO | CTB ALS IgA | 16 | 315.1 (108.5-647.5) | 453.5 (225.5-1016.2) | 0.633 |
| Baseline MPO | CTB fecal IgA | 8 | 282.6 (108.5-521.5) | 418.1 (225.5-1016.2) | 0.534 |
| Baseline MPO | CTB fecal IgA | 16 | 304.8 (108.5-521.5) | 408.6 (225.5-1016.2) | 0.836 |
| Baseline MPO | CTB fecal IgA | 32 | 303.5 (108.5-521.5) | 391.6 (225.5-1016.2) | 0.776 |
| Peak MPO | CS17 serum IgA | 2 | 5615.9 (3456-7424.2) | 10688.1 (1178-52500) | 0.18 |
| Peak MPO | CS17 serum IgA | 4 | 5337.7 (1523.7-11383.3) | 13701.7 (1178-52500) | 0.066 |
| Peak MPO | CS17 serum IgA | 8 | 10476.6 (1523.7-52500) | 7561 (1178-25687.5) | 0.679 |
| Peak MPO | CS17 serum IgG | 4 | 10475.2 (5583-22934.6) | 8741.1 (1178-52500) | 1 |
| Peak MPO | CS17 serum IgG | 8 | 12237.5 (5583-52500) | 5541.5 (1178-32794.2) | 0.44 |
| Peak MPO | CS17 ALS IgA | 16 | 4053.1 (1523.7-7424.2) | 12758.8 (1178-52500) | 0.04 |
| Peak MPO | CS17 ALS IgA | 32 | 4982.9 (1523.7-11383.3) | 12905.2 (1178-52500) | 0.075 |
| Peak MPO | CS17 ALS IgA | 64 | 3918.3 (1178-11383.3) | 16837.6 (5583-52500) | 0.008 |
| Peak MPO | CS17 fecal IgA | 8 | 4274.3 (1523.7-7424.2) | 10087.5 (1178-32794.2) | 0.145 |
| Peak MPO | CS17 fecal IgA | 16 | 5284.8 (1523.7-9989.4) | 10992.4 (1178-32794.2) | 0.109 |
| Peak MPO | CS17 fecal IgA | 32 | 5255.6 (1178-22934.6) | 14832 (7528-32794.2) | 0.082 |
| Peak MPO | CTB serum IgA | 2 | 6033.2 (1178-25687.5) | 18267.9 (7424.2-52500) | 0.0496 |
| Peak MPO | CTB serum IgG | 2 | 8139.6 (1523.7-25687.5) | 11657.5 (1178-52500) | 0.456 |
| Peak MPO | CTB ALS IgA | 4 | 8725.1 (1523.7-32794.2) | 9874 (1178-52500) | 0.776 |
| Peak MPO | CTB ALS IgA | 8 | 8731.1 (1523.7-32794.2) | 10493.3 (1178-52500) | 0.689 |
| Peak MPO | CTB ALS IgA | 16 | 8172.1 (1178-32794.2) | 16432.1 (7424.2-52500) | 0.448 |
| Peak MPO | CTB fecal IgA | 8 | 7387.5 (1178-32794.2) | 11541.3 (5583-52500) | 0.836 |
| Peak MPO | CTB fecal IgA | 16 | 7712.9 (1178-32794.2) | 11822.5 (5583-52500) | 0.836 |
| Peak MPO | CTB fecal IgA | 32 | 7689.6 (1178-32794.2) | 10736.1 (7424.2-14642.6) | 0.921 |
| Peak MPO fold change | CS17 serum IgA | 2 | 12.9 (7.3-21.5) | 33.6 (2.3-160.8) | 0.233 |
| Peak MPO fold change | CS17 serum IgA | 4 | 15.9 (5.8-50.5) | 40.2 (2.3-160.8) | 0.113 |
| Peak MPO fold change | CS17 serum IgA | 8 | 34.4 (5.8-160.8) | 18 (2.3-83.1) | 0.44 |
| Peak MPO fold change | CS17 serum IgG | 4 | 37.1 (8.6-160.8) | 22.8 (2.3-129) | 0.529 |
| Peak MPO fold change | CS17 serum IgG | 8 | 35.1 (7.3-160.8) | 17.3 (2.3-105.4) | 0.44 |
| Peak MPO fold change | CS17 ALS IgA | 16 | 10.5 (5.8-21.5) | 39.4 (2.3-160.8) | 0.056 |
| Peak MPO fold change | CS17 ALS IgA | 32 | 14.4 (5.8-50.5) | 38.5 (2.3-160.8) | 0.099 |
| Peak MPO fold change | CS17 ALS IgA | 64 | 10.6 (2.3-50.5) | 52.7 (8.6-160.8) | 0.012 |
| Peak MPO fold change | CS17 fecal IgA | 8 | 9.7 (5.8-21.5) | 31.8 (2.3-160.8) | 0.145 |
| Peak MPO fold change | CS17 fecal IgA | 16 | 11.7 (5.8-21.5) | 40.7 (2.3-160.8) | 0.073 |
| Peak MPO fold change | CS17 fecal IgA | 32 | 11.9 (2.3-67.4) | 65.7 (25.6-160.8) | 0.03 |
| Peak MPO fold change | CTB serum IgA | 2 | 16.6 (2.3-83.1) | 59.6 (7.3-160.8) | 0.066 |
| Peak MPO fold change | CTB serum IgG | 2 | 26.7 (5.8-160.8) | 29.3 (2.3-129) | 0.864 |
| Peak MPO fold change | CTB ALS IgA | 4 | 23.2 (5.8-105.4) | 31.2 (2.3-160.8) | 0.607 |
| Peak MPO fold change | CTB ALS IgA | 8 | 25.5 (5.8-105.4) | 31.4 (2.3-160.8) | 0.689 |
| Peak MPO fold change | CTB ALS IgA | 16 | 25.9 (2.3-160.8) | 36.2 (7.3-129) | 0.84 |
| Peak MPO fold change | CTB fecal IgA | 8 | 26.1 (2.3-160.8) | 27.6 (7.3-129) | 0.945 |
| Peak MPO fold change | CTB fecal IgA | 16 | 25.3 (2.3-160.8) | 28.9 (7.3-129) | 0.945 |
| Peak MPO fold change | CTB fecal IgA | 32 | 25.3 (2.3-160.8) | 27.4 (7.3-55.9) | 1 |
| Data were displayed as geometric mean (range). MPO concentrations were shown as ng/g. Antibody fold change cut off: cut off of antibody titers fold change from baseline to peak. | | | | | |
