# Supplementary material for: Intestinal inflammation induced by heat-labile toxin-producing enterotoxigenic E: Coli infection and impact on immune responses in an experimental human challenge model
Source: PLoS Negl Trop Dis. 2025 Oct 3;19(10):e0013025. doi: 10.1371/journal.pntd.0013025 (PMC12510637; doi:10.1371/journal.pntd.0013025)
Supplement: S4 Table — (DOCX) [file pntd.0013025.s005.docx]

**Supplemental Table 4**. Non-significant associations between cytokines and immune responses to ETEC vaccine-specific antigens

|  |  |  | **Seroconversion status** | |  |
| --- | --- | --- | --- | --- | --- |
| **Cytokine** | **Antibody** | **Antibody fold change cutoff** | **Non-responder** | **Responder** | **P Value** |
| Baseline IL-1β | CS17 serum IgA | 2 | 1.3 (0.2-18.2) | 1.4 (0.2-9.6) | 0.876 |
| Baseline IL-1β | CS17 serum IgG | 4 | 1.4 (0.3-4.8) | 1.4 (0.2-18.2) | 0.947 |
| Baseline IL-1β | CS17 ALS IgA | 16 | 2.1 (0.2-18.2) | 1.2 (0.2-6.9) | 0.62 |
| Baseline IL-1β | CS17 fecal IgA | 8 | 1.0 (0.2-9.6) | 1.2 (0.3-4.8) | 0.759 |
| Baseline IL-1β | CTB serum IgA | 2 | 1.7 (0.2-18.2) | 1.0 (0.2-6.9) | 0.739 |
| Baseline IL-1β | CTB serum IgG | 2 | 1.7 (0.2-18.2) | 1.1 (0.2-6.9) | 0.698 |
| Baseline IL-1β | CTB ALS IgA | 4 | 1.4 (0.3-9.6) | 1.4 (0.2-18.2) | 0.897 |
| Baseline IL-1β | CTB fecal IgA | 8 | 2.0 (0.5–9.6) | 1.0 (0.2–6.9) | 0.329 |
| Baseline IL-2 | CS17 serum IgA | 2 | 0.8 (0.6-1.3) | 1.1 (0.6-2.2) | 0.266 |
| Baseline IL-2 | CS17 serum IgG | 4 | 1.0 (0.6-1.8) | 1.0 (0.6-2.2) | 1 |
| Baseline IL-2 | CS17 ALS IgA | 16 | 0.9 (0.6-1.4) | 1.1 (0.6-2.2) | 0.564 |
| Baseline IL-2 | CS17 fecal IgA | 8 | 0.8 (0.6-1.4) | 1.0 (0.6-1.8) | 0.597 |
| Baseline IL-2 | CTB serum IgA | 2 | 1.1 (0.6-2.2) | 0.9 (0.6-1.8) | 0.838 |
| Baseline IL-2 | CTB serum IgG | 2 | 1.1 (0.6-2.2) | 0.9 (0.6-1.8) | 0.598 |
| Baseline IL-2 | CTB ALS IgA | 4 | 1.0 (0.6-1.4) | 1.0 (0.6-2.2) | 0.792 |
| Baseline IL-2 | CTB fecal IgA | 8 | 0.9 (0.6–1.4) | 1.0 (0.6–1.8) | 0.502 |
| Baseline IL-4 | CS17 serum IgA | 2 | 0.2 (0.1-0.7) | 0.4 (0.1-0.7) | 0.637 |
| Baseline IL-4 | CS17 serum IgG | 4 | 0.2 (0.1-0.6) | 0.4 (0.1-0.7) | 0.138 |
| Baseline IL-4 | CS17 ALS IgA | 16 | 0.2 (0.1-0.7) | 0.4 (0.1-0.7) | 0.617 |
| Baseline IL-4 | CS17 fecal IgA | 8 | 0.3 (0.1-0.7) | 0.3 (0.1-0.7) | 0.918 |
| Baseline IL-4 | CTB serum IgA | 2 | 0.3 (0.1-0.7) | 0.4 (0.1-0.7) | 0.946 |
| Baseline IL-4 | CTB serum IgG | 2 | 0.3 (0.1-0.7) | 0.4 (0.1-0.7) | 0.602 |
| Baseline IL-4 | CTB ALS IgA | 4 | 0.3 (0.1-0.7) | 0.4 (0.1-0.7) | 0.648 |
| Baseline IL-4 | CTB fecal IgA | 8 | 0.3 (0.1–0.7) | 0.4 (0.1–0.7) | 0.624 |
| Baseline IL-6 | CS17 serum IgA | 2 | 0.9 (0.4-5.3) | 0.8 (0.4-5.3) | 0.633 |
| Baseline IL-6 | CS17 serum IgG | 4 | 0.4 (0.4-0.5) | 1.2 (0.4-5.3) | 0.088 |
| Baseline IL-6 | CS17 ALS IgA | 16 | 0.8 (0.4-5.3) | 0.9 (0.4-5.3) | 0.563 |
| Baseline IL-6 | CS17 fecal IgA | 8 | 1.0 (0.4-5.3) | 0.7 (0.4-4.7) | 1 |
| Baseline IL-6 | CTB serum IgA | 2 | 0.9 (0.4-5.3) | 0.8 (0.4-5.3) | 0.585 |
| Baseline IL-6 | CTB serum IgG | 2 | 0.7 (0.4-5.3) | 1.1 (0.4-5.3) | 0.262 |
| Baseline IL-6 | CTB ALS IgA | 4 | 0.6 (0.4-1.7) | 1.2 (0.4-5.3) | 0.235 |
| Baseline IL-6 | CTB fecal IgA | 8 | 0.7 (0.4–4.7) | 0.8 (0.4–5.3) | 0.318 |
| Baseline IL-8 | CS17 serum IgA | 2 | 0.9 (0.2-7.7) | 2.9 (0.4-35.7) | 0.368 |
| Baseline IL-8 | CS17 serum IgG | 4 | 2.2 (0.4-35.7) | 2.3 (0.2-10.6) | 0.898 |
| Baseline IL-8 | CS17 ALS IgA | 16 | 1.4 (0.2-7.7) | 2.8 (0.4-35.7) | 0.733 |
| Baseline IL-8 | CS17 fecal IgA | 8 | 0.8 (0.2-4.8) | 2.5 (0.4-35.7) | 0.497 |
| Baseline IL-8 | CTB serum IgA | 2 | 3.3 (0.4-35.7) | 1.2 (0.2-10.6) | 0.112 |
| Baseline IL-8 | CTB serum IgG | 2 | 3.5 (0.4-35.7) | 1.3 (0.2-10.6) | 0.142 |
| Baseline IL-8 | CTB ALS IgA | 4 | 3.2 (0.4-35.7) | 1.8 (0.2-10.6) | 0.414 |
| Baseline IL-8 | CTB fecal IgA | 8 | 3.0 (0.4–35.7) | 1.6 (0.2–10.6) | 0.53 |
| Baseline IL-10 | CS17 serum IgA | 2 | 0.3 (0.1-2.5) | 0.8 (0.1-2.8) | 0.434 |
| Baseline IL-10 | CS17 serum IgG | 4 | 0.4 (0.1-1.1) | 0.9 (0.1-2.8) | 0.18 |
| Baseline IL-10 | CS17 ALS IgA | 16 | 0.4 (0.1-2.5) | 0.8 (0.1-2.8) | 0.435 |
| Baseline IL-10 | CS17 fecal IgA | 8 | 0.7 (0.1-2.5) | 0.9 (0.1-2.8) | 0.838 |
| Baseline IL-10 | CTB serum IgA | 2 | 0.7 (0.1-2.8) | 0.7 (0.2-2.8) | 1 |
| Baseline IL-10 | CTB serum IgG | 2 | 0.5 (0.1-2.1) | 0.9 (0.2-2.8) | 0.399 |
| Baseline IL-10 | CTB ALS IgA | 4 | 0.7 (0.1-1.8) | 0.7 (0.1-2.8) | 0.746 |
| Baseline IL-10 | CTB fecal IgA | 8 | 0.7 (0.1–2.8) | 0.7 (0.1–2.5) | 0.935 |
| Baseline IL-13 | CS17 serum IgA | 2 | 4.8 (3.2-11.3) | 12.4 (3.2-25.6) | 0.085 |
| Baseline IL-13 | CS17 serum IgG | 4 | 8.3 (3.2-20.6) | 11.3 (3.2-25.6) | 0.688 |
| Baseline IL-13 | CS17 ALS IgA | 16 | 5.4 (3.2-11.3) | 13.0 (3.2-25.6) | 0.055 |
| Baseline IL-13 | CS17 fecal IgA | 8 | 6.5 (3.2-11.3) | 12.4 (3.2-25.6) | 0.152 |
| Baseline IL-13 | CTB serum IgA | 2 | 9.8 (3.2-25.6) | 10.8 (7.1-16.3) | 0.789 |
| Baseline IL-13 | CTB serum IgG | 2 | 8.6 (3.2-25.0) | 12.5 (7.1-25.6) | 0.65 |
| Baseline IL-13 | CTB ALS IgA | 4 | 10.6 (3.2-21.2) | 9.8 (3.2-25.6) | 0.846 |
| Baseline IL-13 | CTB fecal IgA | 8 | 10.8 (3.2–25.6) | 10.0 (3.2–21.2) | 0.807 |
| Baseline IL-17A | CS17 serum IgG | 4 | 2.6 (1.6-7.7) | 2.4 (1.2-6.2) | 1 |
| Baseline IL-17A | CS17 ALS IgA | 16 | 1.6 (1.2-2.4) | 2.9 (1.2-7.7) | 0.087 |
| Baseline IL-17A | CS17 fecal IgA | 8 | 1.7 (1.2-2.4) | 3.1 (1.6-7.7) | 0.099 |
| Baseline IL-17A | CTB serum IgA | 2 | 2.6 (1.6-7.7) | 2.2 (1.2-6.2) | 0.686 |
| Baseline IL-17A | CTB serum IgG | 2 | 2.6 (1.6-7.7) | 2.3 (1.2-6.2) | 0.557 |
| Baseline IL-17A | CTB ALS IgA | 4 | 2.8 (1.6-7.7) | 2.2 (1.2-6.2) | 0.215 |
| Baseline IL-17A | CTB fecal IgA | 8 | 2.8 (1.6–7.7) | 2.2 (1.2–6.2) | 0.566 |
| Baseline TNF-α | CS17 serum IgA | 2 | 1.6 (1.1-3.9) | 2.3 (0.9-7.2) | 0.524 |
| Baseline TNF-α | CS17 serum IgG | 4 | 1.4 (1.1-1.8) | 2.6 (0.9-7.2) | 0.306 |
| Baseline TNF-α | CS17 ALS IgA | 16 | 2.0 (1.1-3.9) | 2.1 (0.9-7.2) | 0.828 |
| Baseline TNF-α | CS17 fecal IgA | 8 | 2.5 (1.1-3.9) | 2.1 (1.1-7.2) | 0.916 |
| Baseline TNF-α | CTB serum IgA | 2 | 2.4 (1.1-7.2) | 1.6 (0.9-3.9) | 0.375 |
| Baseline TNF-α | CTB serum IgG | 2 | 2.1 (1.1-5.8) | 2.1 (0.9-7.2) | 1 |
| Baseline TNF-α | CTB ALS IgA | 4 | 2.1 (1.1-5.8) | 2.1 (0.9-7.2) | 1 |
| Baseline TNF-α | CTB fecal IgA | 8 | 2.2 (1.1–7.2) | 2.0 (0.9–5.8) | 0.934 |
| Baseline IFN-γ | CS17 serum IgA | 2 | 15.1 (9.8-35.4) | 12.9 (4.1-40.3) | 0.752 |
| Baseline IFN-γ | CS17 serum IgG | 4 | 12.2 (8.8-36.6) | 14.0 (4.1-40.3) | 0.498 |
| Baseline IFN-γ | CS17 ALS IgA | 16 | 13.2 (8.8-35.4) | 13.4 (4.1-40.3) | 0.829 |
| Baseline IFN-γ | CS17 fecal IgA | 8 | 14.5 (8.8-35.4) | 15.9 (8.8-40.3) | 0.755 |
| Baseline IFN-γ | CTB serum IgA | 2 | 13.1 (8.8-36.6) | 13.8 (4.1-40.3) | 0.946 |
| Baseline IFN-γ | CTB serum IgG | 2 | 12.2 (8.8-36.6) | 15.0 (4.1-40.3) | 0.793 |
| Baseline IFN-γ | CTB ALS IgA | 4 | 12.8 (8.8-36.6) | 13.7 (4.1-40.3) | 0.743 |
| Baseline IFN-γ | CTB fecal IgA | 8 | 11.2 (8.8–23.2) | 16.2 (4.1–40.3) | 0.41 |
| Peak IL-1β | CS17 serum IgA | 2 | 98.1 (59.4-188.1) | 674.8 (43.4-4288.6) | 0.126 |
| Peak IL-1β | CS17 serum IgG | 4 | 236.2 (59.4-1563.4) | 635.6 (43.4-4288.6) | 0.364 |
| Peak IL-1β | CTB serum IgA | 2 | 270.4 (43.4-1872.0) | 1100.4 (84.4-4288.6) | 0.112 |
| Peak IL-1β | CTB serum IgG | 2 | 240.1 (43.4-1872.0) | 1020.5 (84.4-4288.6) | 0.108 |
| Peak IL-1β | CTB ALS IgA | 4 | 209.3 (43.4-4195.1) | 787.7 (84.4-4288.6) | 0.081 |
| Peak IL-1β | CTB fecal IgA | 8 | 257.6 (43.4–4195.1) | 609.4 (74.8–4288.6) | 0.343 |
| Peak IL-2 | CS17 serum IgA | 2 | 3.4 (1.3-20.5) | 4.9 (2.2-13.8) | 0.456 |
| Peak IL-2 | CS17 serum IgG | 4 | 6.4 (2.8-20.5) | 3.7 (1.3-13.8) | 0.364 |
| Peak IL-2 | CS17 ALS IgA | 16 | 3.7 (1.3-20.5) | 4.8 (2.2-13.8) | 0.733 |
| Peak IL-2 | CS17 fecal IgA | 8 | 5.3 (1.5-20.5) | 5.8 (2.8-13.8) | 0.921 |
| Peak IL-2 | CTB serum IgA | 2 | 4.3 (1.3-20.5) | 4.9 (1.5-13.8) | 0.898 |
| Peak IL-2 | CTB serum IgG | 2 | 4.4 (1.3-20.5) | 4.6 (1.5-13.8) | 1 |
| Peak IL-2 | CTB fecal IgA | 8 | 8.2 (3.0–20.5) | 3.9 (1.5–11.1) | 0.073 |
| Peak IL-4 | CS17 serum IgA | 2 | 0.5 (0.1-1.0) | 0.9 (0.2-2.2) | 0.225 |
| Peak IL-4 | CS17 serum IgG | 4 | 0.9 (0.3-2.2) | 0.7 (0.1-1.7) | 0.797 |
| Peak IL-4 | CS17 ALS IgA | 16 | 0.6 (0.1-1.6) | 0.8 (0.2-2.2) | 0.635 |
| Peak IL-4 | CS17 fecal IgA | 8 | 1.0 (0.7-1.6) | 0.9 (0.2-2.2) | 0.921 |
| Peak IL-4 | CTB serum IgA | 2 | 0.8 (0.1-2.2) | 0.8 (0.2-1.7) | 1 |
| Peak IL-4 | CTB serum IgG | 2 | 0.7 (0.1-2.2) | 0.9 (0.2-1.7) | 0.755 |
| Peak IL-4 | CTB ALS IgA | 4 | 0.9 (0.2-2.2) | 0.7 (0.1-1.7) | 0.662 |
| Peak IL-4 | CTB fecal IgA | 8 | 0.8 (0.2–1.6) | 1.0 (0.3–2.2) | 0.53 |
| Peak IL-6 | CS17 serum IgA | 2 | 1.5 (0.4-6.3) | 3.2 (1.1-9.2) | 0.456 |
| Peak IL-6 | CS17 serum IgG | 4 | 2.2 (1.1-7.3) | 3.1 (0.4-9.2) | 0.438 |
| Peak IL-6 | CS17 ALS IgA | 16 | 1.8 (0.4-6.3) | 3.2 (1.1-9.2) | 0.374 |
| Peak IL-6 | CS17 fecal IgA | 8 | 3.0 (1.5-6.3) | 3.4 (1.1-9.2) | 0.63 |
| Peak IL-6 | CTB serum IgA | 2 | 2.6 (0.4-7.3) | 3.0 (1.1-9.2) | 0.898 |
| Peak IL-6 | CTB serum IgG | 2 | 2.4 (0.4-7.3) | 3.3 (1.1-9.2) | 0.573 |
| Peak IL-6 | CTB ALS IgA | 4 | 2.8 (1.5-7.3) | 2.7 (0.4-9.2) | 1 |
| Peak IL-6 | CTB fecal IgA | 8 | 2.5 (1.5–5.4) | 3.5 (1.1–9.2) | 0.53 |
| Peak IL-8 | CS17 serum IgA | 2 | 38.0 (31.9-48.5) | 197.2 (17.7-7663.6) | 0.555 |
| Peak IL-8 | CS17 serum IgG | 4 | 124.0 (23.5-1124.7) | 147.4 (17.7-7663.6) | 0.898 |
| Peak IL-8 | CS17 ALS IgA | 16 | 31.4 (17.7-48.5) | 250.9 (20.0-7663.6) | 0.142 |
| Peak IL-8 | CS17 fecal IgA | 8 | 30.2 (17.7-48.5) | 285.6 (20.0-7663.6) | 0.133 |
| Peak IL-8 | CTB serum IgG | 2 | 59.5 (17.7-1124.7) | 427.5 (20.0-7663.6) | 0.142 |
| Peak IL-8 | CTB ALS IgA | 4 | 106.7 (17.7-7663.6) | 168.6 (20.0-2155.1) | 0.662 |
| Peak IL-8 | CTB fecal IgA | 8 | 97.0 (17.7–7663.6) | 285.2 (23.5–2155.1) | 0.268 |
| Peak IL-10 | CS17 serum IgA | 2 | 0.7 (0.1-2.9) | 2.1 (0.8-3.4) | 0.368 |
| Peak IL-10 | CS17 serum IgG | 4 | 1.8 (0.8-2.7) | 1.6 (0.1-3.4) | 0.606 |
| Peak IL-10 | CS17 ALS IgA | 16 | 0.9 (0.1-2.9) | 2.1 (0.8-3.4) | 0.188 |
| Peak IL-10 | CS17 fecal IgA | 8 | 1.6 (0.8-2.9) | 2.4 (1.8-3.4) | 0.279 |
| Peak IL-10 | CTB serum IgA | 2 | 1.4 (0.1-2.8) | 2.2 (0.8-3.4) | 0.112 |
| Peak IL-10 | CTB ALS IgA | 4 | 1.9 (0.8-2.9) | 1.5 (0.1-3.4) | 0.852 |
| Peak IL-10 | CTB fecal IgA | 8 | 2.0 (0.8–2.9) | 2.0 (0.8–3.4) | 1 |
| Peak IL-13 | CS17 serum IgA | 2 | 9.0 (3.2-23.7) | 16.1 (5.6-35.3) | 0.291 |
| Peak IL-13 | CS17 serum IgG | 4 | 9.3 (3.2-20.6) | 17.9 (7.7-35.3) | 0.147 |
| Peak IL-13 | CS17 ALS IgA | 16 | 8.6 (3.2-23.7) | 17.3 (5.6-35.3) | 0.106 |
| Peak IL-13 | CS17 fecal IgA | 8 | 8.3 (3.2-23.7) | 16.8 (5.6-35.3) | 0.279 |
| Peak IL-13 | CTB serum IgA | 2 | 12.1 (3.2-28.1) | 18.7 (11.4-35.3) | 0.438 |
| Peak IL-13 | CTB serum IgG | 2 | 11.1 (3.2-28.1) | 19.7 (11.4-35.3) | 0.181 |
| Peak IL-13 | CTB ALS IgA | 4 | 11.1 (3.2-28.1) | 17.0 (5.6-35.3) | 0.345 |
| Peak IL-13 | CTB fecal IgA | 8 | 10.8 (3.2–25.6) | 16.8 (5.6–35.3) | 0.343 |
| Peak IL-17A | CS17 serum IgG | 4 | 5.7 (1.6-8.1) | 7.3 (1.6-42.5) | 0.947 |
| Peak IL-17A | CS17 ALS IgA | 16 | 3.6 (1.6-9.4) | 8.6 (3.7-42.5) | 0.229 |
| Peak IL-17A | CS17 fecal IgA | 8 | 4.8 (1.6-9.4) | 10.4 (6.3-42.5) | 0.376 |
| Peak IL-17A | CTB serum IgA | 2 | 5.2 (1.6-9.4) | 10.8 (3.7-42.5) | 0.423 |
| Peak IL-17A | CTB serum IgG | 2 | 5.0 (1.6-9.4) | 9.8 (3.7-42.5) | 0.651 |
| Peak IL-17A | CTB ALS IgA | 4 | 7.0 (1.6-15.9) | 6.5 (1.6-42.5) | 0.155 |
| Peak IL-17A | CTB fecal IgA | 8 | 6.5 (1.6–15.9) | 8.9 (3.7–42.5) | 0.876 |
| Peak TNF-α | CS17 serum IgA | 2 | 3.8 (1.1-7.9) | 6.5 (2.8-19.4) | 0.659 |
| Peak TNF-α | CS17 serum IgG | 4 | 6.5 (3.2-19.4) | 5.4 (1.1-15.0) | 1 |
| Peak TNF-α | CS17 ALS IgA | 16 | 3.8 (1.1-7.9) | 6.8 (2.8-19.4) | 0.374 |
| Peak TNF-α | CS17 fecal IgA | 8 | 5.9 (4.0-7.9) | 7.8 (3.2-19.4) | 0.497 |
| Peak TNF-α | CTB serum IgA | 2 | 5.0 (1.1-19.4) | 7.4 (2.8-15.0) | 0.298 |
| Peak TNF-α | CTB serum IgG | 2 | 4.8 (1.1-19.4) | 7.4 (2.8-15.0) | 0.181 |
| Peak TNF-α | CTB ALS IgA | 4 | 5.8 (3.2-15.0) | 5.7 (1.1-19.4) | 0.662 |
| Peak TNF-α | CTB fecal IgA | 8 | 6.6 (4.0–15.0) | 6.7 (2.8–19.4) | 0.876 |
| Peak IFN-γ | CS17 serum IgA | 2 | 15.5 (9.8-38.8) | 34.2 (10.9-138.6) | 0.119 |
| Peak IFN-γ | CS17 serum IgG | 4 | 29.9 (9.8-43.3) | 28.3 (9.8-138.6) | 0.947 |
| Peak IFN-γ | CS17 ALS IgA | 16 | 20.0 (9.8-43.0) | 33.4 (10.9-138.6) | 0.357 |
| Peak IFN-γ | CS17 fecal IgA | 8 | 25.4 (9.8-43.0) | 40.3 (16.5-138.6) | 0.63 |
| Peak IFN-γ | CTB serum IgA | 2 | 24.6 (9.8-47.0) | 38.4 (16.5-138.6) | 0.593 |
| Peak IFN-γ | CTB serum IgG | 2 | 24.3 (9.8-47.0) | 36.2 (16.5-138.6) | 0.747 |
| Peak IFN-γ | CTB ALS IgA | 4 | 28.4 (9.8-47.0) | 29.2 (9.8-138.6) | 0.605 |
| Peak IFN-γ | CTB fecal IgA | 8 | 24.1 (9.8–43.3) | 44.0 (22.5–138.6) | 0.343 |
| Peak IL-1β fold change | CS17 serum IgA | 2 | 76.4 (10.3-357.8) | 468.7 (4.5-15258.9) | 0.225 |
| Peak IL-1β fold change | CS17 serum IgG | 4 | 167.3 (31.1-399.0) | 453.8 (4.5-15258.9) | 0.298 |
| Peak IL-1β fold change | CS17 fecal IgA | 8 | 57.9 (4.5-357.8) | 625.3 (31.1-15258.9) | 0.133 |
| Peak IL-1β fold change | CTB serum IgA | 2 | 163.2 (4.5-10508.9) | 1053.6 (216.9-15258.9) | 0.298 |
| Peak IL-1β fold change | CTB serum IgG | 2 | 139.7 (4.5-10508.9) | 950.3 (216.9-15258.9) | 0.181 |
| Peak IL-1β fold change | CTB ALS IgA | 4 | 149.5 (4.5-3331.1) | 559.4 (10.3-15258.9) | 0.345 |
| Peak IL-1β fold change | CTB fecal IgA | 8 | 126.1 (4.5–3331.1) | 608.5 (216.9–15258.9) | 0.343 |
| Peak IL-2 fold change | CS17 serum IgA | 2 | 4.3 (1-35.3) | 4.4 (1-23.9) | 0.585 |
| Peak IL-2 fold change | CS17 serum IgG | 4 | 6.1 (2.7-35.3) | 3.7 (1.0-23.9) | 0.423 |
| Peak IL-2 fold change | CS17 ALS IgA | 16 | 4.1 (1-35.3) | 4.6 (1-23.9) | 0.479 |
| Peak IL-2 fold change | CS17 fecal IgA | 8 | 6.5 (2.3-35.3) | 5.6 (2.7-23.9) | 0.776 |
| Peak IL-2 fold change | CTB serum IgA | 2 | 4.0 (1-35.3) | 5.4 (2.3-23.9) | 0.894 |
| Peak IL-2 fold change | CTB serum IgG | 2 | 3.9 (1-35.3) | 5.3 (2.3-23.9) | 0.747 |
| Peak IL-2 fold change | CTB ALS IgA | 4 | 8.0 (3.1-35.3) | 2.8 (1.0-7.7) | 0.137 |
| Peak IL-2 fold change | CTB fecal IgA | 8 | 9.6 (3.4–35.3) | 3.9 (2.3–7.7) | 0.106 |
| Peak IL-4 fold change | CS17 serum IgA | 2 | 2.9 (1.4-9.4) | 2.3 (1-5.2) | 0.755 |
| Peak IL-4 fold change | CS17 ALS IgA | 16 | 2.9 (1.4-9.4) | 2.2 (1-5.2) | 0.62 |
| Peak IL-4 fold change | CS17 fecal IgA | 8 | 3.3 (1.4-9.4) | 2.7 (1.4-5.2) | 0.921 |
| Peak IL-4 fold change | CTB serum IgA | 2 | 2.5 (1-9.4) | 2.2 (1-4.3) | 0.841 |
| Peak IL-4 fold change | CTB serum IgG | 2 | 2.6 (1-9.4) | 2.2 (1-4.3) | 0.698 |
| Peak IL-4 fold change | CTB ALS IgA | 4 | 3.1 (1.4-9.4) | 2.0 (1.0-4.3) | 0.272 |
| Peak IL-4 fold change | CTB fecal IgA | 8 | 2.9 (1.4–9.4) | 2.4 (1.0–5.2) | 0.876 |
| Peak IL-6 fold change | CS17 serum IgA | 2 | 1.7 (1-3.9) | 3.8 (1-18.4) | 0.242 |
| Peak IL-6 fold change | CS17 serum IgG | 4 | 4.9 (2.2-14.5) | 2.5 (1.0-18.4) | 0.182 |
| Peak IL-6 fold change | CS17 ALS IgA | 16 | 2.3 (1-5.7) | 3.7 (1-18.4) | 0.479 |
| Peak IL-6 fold change | CS17 fecal IgA | 8 | 3.0 (1.2-5.7) | 4.7 (1.1-18.4) | 0.63 |
| Peak IL-6 fold change | CTB serum IgA | 2 | 3.0 (1-14.5) | 3.7 (1.2-18.4) | 0.593 |
| Peak IL-6 fold change | CTB serum IgG | 2 | 3.3 (1-14.5) | 3.0 (1.1-18.4) | 1 |
| Peak IL-6 fold change | CTB ALS IgA | 4 | 5.0 (2.0-14.5) | 2.3 (1.0-18.4) | 0.106 |
| Peak IL-6 fold change | CTB fecal IgA | 8 | 3.6 (1.1–7.5) | 4.1 (1.2–18.4) | 1 |
| Peak IL-8 fold change | CS17 serum IgA | 2 | 40.8 (4.6-205.5) | 67.7 (2.8-5397.6) | 0.885 |
| Peak IL-8 fold change | CS17 serum IgG | 4 | 55.8 (2.8-801.0) | 63.6 (3.7-5397.6) | 0.898 |
| Peak IL-8 fold change | CS17 ALS IgA | 16 | 22.4 (3.7-205.5) | 90.5 (2.8-5397.6) | 0.374 |
| Peak IL-8 fold change | CS17 fecal IgA | 8 | 37.8 (3.7-205.5) | 116.3 (2.8-5397.6) | 0.63 |
| Peak IL-8 fold change | CTB ALS IgA | 4 | 33.0 (2.8-5397.6) | 95.9 (4.6-1657.5) | 0.282 |
| Peak IL-8 fold change | CTB fecal IgA | 8 | 32.3 (2.8–5397.6) | 175.9 (6.0–1657.5) | 0.268 |
| Peak IL-10 fold change | CS17 serum IgA | 2 | 2.0 (1-6.6) | 2.6 (1-14.3) | 0.639 |
| Peak IL-10 fold change | CS17 serum IgG | 4 | 4.2 (2.0-14.3) | 1.8 (1.0-8.4) | 0.082 |
| Peak IL-10 fold change | CS17 ALS IgA | 16 | 2.0 (1-6.6) | 2.6 (1-14.3) | 0.619 |
| Peak IL-10 fold change | CS17 fecal IgA | 8 | 2.5 (1.1-6.6) | 2.8 (1.0-14.3) | 0.921 |
| Peak IL-10 fold change | CTB serum IgA | 2 | 2.1 (1-14.3) | 3.0 (1.1-8.4) | 0.228 |
| Peak IL-10 fold change | CTB serum IgG | 2 | 2.4 (1-14.3) | 2.5 (1-8.4) | 0.65 |
| Peak IL-10 fold change | CTB ALS IgA | 4 | 2.9 (1.1-8.4) | 2.1 (1.0-14.3) | 0.476 |
| Peak IL-10 fold change | CTB fecal IgA | 8 | 3.1 (1.0–8.4) | 2.6 (1.1–14.3) | 0.876 |
| Peak IL-13 fold change | CS17 serum IgA | 2 | 1.9 (1-3.1) | 1.3 (1-5.0) | 0.262 |
| Peak IL-13 fold change | CS17 serum IgG | 4 | 1.1 (1.0-1.8) | 1.6 (1.0-5.0) | 0.237 |
| Peak IL-13 fold change | CS17 ALS IgA | 16 | 1.6 (1-3.1) | 1.3 (1-5.0) | 0.584 |
| Peak IL-13 fold change | CS17 fecal IgA | 8 | 1.3 (1.0-2.1) | 1.4 (1.0-5.0) | 1 |
| Peak IL-13 fold change | CTB serum IgA | 2 | 1.2 (1-3.1) | 1.7 (1-5.0) | 0.301 |
| Peak IL-13 fold change | CTB serum IgG | 2 | 1.3 (1-3.1) | 1.6 (1-5.0) | 0.567 |
| Peak IL-13 fold change | CTB ALS IgA | 4 | 1.0 (1.0-1.3) | 1.7 (1.0-5.0) | 0.063 |
| Peak IL-17A fold change | CS17 serum IgA | 2 | 1.8 (1-6.0) | 3.0 (1-6.8) | 0.348 |
| Peak IL-17A fold change | CS17 serum IgG | 4 | 2.2 (1.0-4.9) | 3.0 (1.0-6.8) | 0.688 |
| Peak IL-17A fold change | CS17 ALS IgA | 16 | 2.2 (1-6.0) | 3.0 (1-6.8) | 0.619 |
| Peak IL-17A fold change | CS17 fecal IgA | 8 | 2.9 (1.0-6.0) | 3.4 (1.0-6.8) | 0.921 |
| Peak IL-17A fold change | CTB serum IgG | 2 | 1.9 (1-4.9) | 4.3 (2.6-6.8) | 0.08 |
| Peak IL-17A fold change | CTB ALS IgA | 4 | 2.5 (1.0-6.4) | 2.9 (1.0-6.8) | 0.948 |
| Peak IL-17A fold change | CTB fecal IgA | 8 | 2.3 (1.0–6.4) | 4.0 (2.7–6.8) | 0.268 |
| Peak TNF-α fold change | CS17 serum IgA | 2 | 2.3 (1-5.9) | 2.9 (1-18.3) | 0.754 |
| Peak TNF-α fold change | CS17 serum IgG | 4 | 4.6 (1.8-18.3) | 2.1 (1.0-14.1) | 0.141 |
| Peak TNF-α fold change | CS17 ALS IgA | 16 | 1.9 (1-5.9) | 3.2 (1-18.3) | 0.435 |
| Peak TNF-α fold change | CS17 fecal IgA | 8 | 2.3 (1.1-5.9) | 3.6 (1.0-18.3) | 0.63 |
| Peak TNF-α fold change | CTB serum IgA | 2 | 2.0 (1-18.3) | 4.6 (2.0-14.1) | 0.082 |
| Peak TNF-α fold change | CTB serum IgG | 2 | 2.2 (1-18.3) | 3.6 (1-14.1) | 0.331 |
| Peak TNF-α fold change | CTB ALS IgA | 4 | 2.8 (1.1-14.1) | 2.7 (1.0-18.3) | 0.846 |
| Peak TNF-α fold change | CTB fecal IgA | 8 | 3.0 (1.0–14.1) | 3.4 (1.1–18.3) | 0.639 |
| Peak IFN-γ fold change | CS17 serum IgA | 2 | 1.0 (1-1.1) | 2.7 (1-5.6) | 0.059 |
| Peak IFN-γ fold change | CS17 serum IgG | 4 | 2.4 (1.0-4.9) | 2.0 (1.0-5.6) | 0.893 |
| Peak IFN-γ fold change | CS17 ALS IgA | 16 | 1.5 (1-4.9) | 2.5 (1-5.6) | 0.283 |
| Peak IFN-γ fold change | CS17 fecal IgA | 8 | 1.8 (1.0-4.9) | 2.5 (1.0-4.9) | 0.539 |
| Peak IFN-γ fold change | CTB serum IgA | 2 | 1.9 (1-4.9) | 2.8 (1.1-5.6) | 0.281 |
| Peak IFN-γ fold change | CTB serum IgG | 2 | 2.0 (1-4.9) | 2.4 (1.1-5.6) | 0.396 |
| Peak IFN-γ fold change | CTB ALS IgA | 4 | 2.2 (1.0-4.9) | 2.1 (1.0-5.6) | 1 |
| Peak IFN-γ fold change | CTB fecal IgA | 8 | 2.2 (1.0–4.9) | 2.7 (1.0–5.6) | 0.935 |
| Data were displayed as geometric mean (range). Cytokine concentrations were shown as ng/g. Antibody fold change cut off: cut off of antibody titers fold change from baseline to peak. | | | | | |

Associations between cytokines and immune responses to ETEC vaccine-specific antigens were analyzed using alternative antibody fold change cutoffs, with results available at the S5 Table.
