# Supplementary material for: Intestinal inflammation induced by heat-labile toxin-producing enterotoxigenic E: Coli infection and impact on immune responses in an experimental human challenge model
Source: PLoS Negl Trop Dis. 2025 Oct 3;19(10):e0013025. doi: 10.1371/journal.pntd.0013025 (PMC12510637; doi:10.1371/journal.pntd.0013025)
Supplement: S5 Table — (DOCX) [file pntd.0013025.s006.docx]

**Supplemental Table 5**. Associations between cytokines and immune responses to ETEC vaccine-specific antigens were analyzed using alternative antibody fold change cutoffs

|  |  |  | **Seroconversion status** | |  |
| --- | --- | --- | --- | --- | --- |
| **Cytokine** | **Antibody** | **Antibody fold change cutoff** | **Non-responder** | **Responder** | **P Value** |
| Baseline IL-1β | CS17 serum IgA | 4 | 2.4 (0.2-18.2) | 1.0 (0.2-6.9) | 0.332 |
| Baseline IL-1β | CS17 serum IgG | 8 | 1.1 (0.2-6.9) | 2.2 (0.2-18.2) | 0.505 |
| Baseline IL-1β | CS17 ALS IgA | 32 | 2.1 (0.2-18.2) | 1.1 (0.2-6.9) | 0.505 |
| Baseline IL-1β | CS17 ALS IgA | 64 | 1.9 (0.2 - 18.2) | 1.1 (0.2 - 6.9) | 0.561 |
| Baseline IL-1β | CS17 fecal IgA | 16 | 1.0 (0.2 - 9.6) | 1.6 (0.3 - 4.8) | 0.476 |
| Baseline IL-1β | CS17 fecal IgA | 32 | 1.4 (0.2-9.6) | 1.3 (0.3-3.9) | 0.914 |
| Baseline IL-1β | CTB ALS IgA | 8 | 1.7 (0.2-18.2) | 1.0 (0.2-6.9) | 0.64 |
| Baseline IL-1β | CTB ALS IgA | 16 | 1.4 (0.2-18.2) | 1.5 (0.2-6.9) | 1 |
| Baseline IL-1β | CTB fecal IgA | 16 | 1.8 (0.5-9.6) | 1.0 (0.2-6.9) | 0.47 |
| Baseline IL-1β | CTB fecal IgA | 32 | 2.0 (0.5-9.6) | 0.5 (0.2-2.1) | 0.183 |
| Baseline IL-2 | CS17 serum IgA | 4 | 0.9 (0.6-1.8) | 1.1 (0.6-2.2) | 0.598 |
| Baseline IL-2 | CS17 serum IgA | 8 | 0.9 (0.6-1.8) | 1.3 (0.7-2.2) | 0.221 |
| Baseline IL-2 | CS17 serum IgG | 8 | 1.0 (0.6-1.8) | 1.1 (0.6-2.2) | 0.838 |
| Baseline IL-2 | CS17 ALS IgA | 32 | 1.0 (0.6-1.8) | 1.0 (0.6-2.2) | 0.946 |
| Baseline IL-2 | CS17 ALS IgA | 64 | 1.0 (0.6 - 1.8) | 1.1 (0.6 - 2.2) | 0.895 |
| Baseline IL-2 | CS17 fecal IgA | 16 | 0.9 (0.6 - 1.4) | 1.0 (0.6 - 1.8) | 0.826 |
| Baseline IL-2 | CS17 fecal IgA | 32 | 0.9 (0.6-1.4) | 1.0 (0.6-1.8) | 1 |
| Baseline IL-2 | CTB ALS IgA | 8 | 1.1 (0.6-2.2) | 0.9 (0.7-1.8) | 0.946 |
| Baseline IL-2 | CTB ALS IgA | 16 | 1.0 (0.6-2.2) | 0.9 (0.7-1.8) | 1 |
| Baseline IL-2 | CTB fecal IgA | 16 | 0.9 (0.6-1.4) | 1.0 (0.6-1.8) | 0.563 |
| Baseline IL-2 | CTB fecal IgA | 32 | 0.8 (0.6-1.4) | 1.2 (0.7-1.8) | 0.197 |
| Baseline IL-4 | CS17 serum IgA | 4 | 0.2 (0.1-0.7) | 0.5 (0.1-0.7) | 0.151 |
| Baseline IL-4 | CS17 serum IgA | 8 | 0.2 (0.1-0.7) | 0.6 (0.4-0.7) | 0.08 |
| Baseline IL-4 | CS17 serum IgG | 8 | 0.4 (0.1-0.7) | 0.3 (0.1-0.7) | 0.787 |
| Baseline IL-4 | CS17 ALS IgA | 32 | 0.2 (0.1-0.7) | 0.4 (0.1-0.7) | 0.419 |
| Baseline IL-4 | CS17 ALS IgA | 64 | 0.3 (0.1 - 0.7) | 0.4 (0.1 - 0.7) | 0.744 |
| Baseline IL-4 | CS17 fecal IgA | 16 | 0.4 (0.1 - 0.7) | 0.3 (0.1 - 0.7) | 0.45 |
| Baseline IL-4 | CS17 fecal IgA | 32 | 0.5 (0.1-0.7) | 0.2 (0.1-0.6) | 0.106 |
| Baseline IL-4 | CTB ALS IgA | 8 | 0.2 (0.1-0.7) | 0.6 (0.3-0.7) | 0.225 |
| Baseline IL-4 | CTB ALS IgA | 16 | 0.3 (0.1-0.7) | 0.5 (0.3-0.7) | 0.529 |
| Baseline IL-4 | CTB fecal IgA | 16 | 0.3 (0.1-0.7) | 0.4 (0.1-0.7) | 0.936 |
| Baseline IL-4 | CTB fecal IgA | 32 | 0.3 (0.1-0.7) | 0.5 (0.3-0.7) | 0.489 |
| Baseline IL-6 | CS17 serum IgA | 4 | 0.6 (0.4-5.3) | 1.1 (0.4-5.3) | 0.187 |
| Baseline IL-6 | CS17 serum IgA | 8 | 0.6 (0.4-5.3) | 1.6 (0.5-5.3) | 0.066 |
| Baseline IL-6 | CS17 serum IgG | 8 | 0.7 (0.4-5.3) | 1.1 (0.4-5.3) | 0.946 |
| Baseline IL-6 | CS17 ALS IgA | 32 | 0.7 (0.4-5.3) | 0.9 (0.4-5.3) | 0.495 |
| Baseline IL-6 | CS17 ALS IgA | 64 | 1.0 (0.4 - 5.3) | 0.8 (0.4 - 5.3) | 0.947 |
| Baseline IL-6 | CS17 fecal IgA | 16 | 1.1 (0.4 - 5.3) | 0.7 (0.4 - 4.7) | 0.585 |
| Baseline IL-6 | CS17 fecal IgA | 32 | 1.3 (0.4-5.3) | 0.4 (0.4-0.5) | 0.229 |
| Baseline IL-6 | CTB ALS IgA | 8 | 0.7 (0.4-5.3) | 1.4 (0.5-5.3) | 0.066 |
| Baseline IL-6 | CTB ALS IgA | 16 | 0.8 (0.4-5.3) | 1.2 (0.5-5.3) | 0.232 |
| Baseline IL-6 | CTB fecal IgA | 16 | 0.8 (0.4-4.7) | 0.8 (0.4-5.3) | 0.681 |
| Baseline IL-6 | CTB fecal IgA | 32 | 0.7 (0.4-4.7) | 1.1 (0.5-5.3) | 0.243 |
| Baseline IL-8 | CS17 serum IgA | 4 | 1.2 (0.2-7.7) | 3.6 (1.3-35.7) | 0.282 |
| Baseline IL-8 | CS17 serum IgA | 8 | 2.3 (0.2-35.7) | 2.3 (1.3-4.0) | 1 |
| Baseline IL-8 | CS17 serum IgG | 8 | 2.0 (0.2-35.7) | 2.9 (1.4-7.7) | 0.606 |
| Baseline IL-8 | CS17 ALS IgA | 32 | 1.1 (0.2-7.7) | 3.4 (1.3-35.7) | 0.298 |
| Baseline IL-8 | CS17 ALS IgA | 64 | 1.3 (0.2 - 7.7) | 3.6 (1.3 - 35.7) | 0.345 |
| Baseline IL-8 | CS17 fecal IgA | 16 | 1.1 (0.2 - 4.8) | 2.2 (0.4 - 35.7) | 0.914 |
| Baseline IL-8 | CS17 fecal IgA | 32 | 2.2 (0.2-35.7) | 1.1 (0.4-2.0) | 0.352 |
| Baseline IL-8 | CTB ALS IgA | 8 | 3.2 (0.4-35.7) | 1.3 (0.2-10.6) | 0.24 |
| Baseline IL-8 | CTB ALS IgA | 16 | 2.8 (0.4-35.7) | 1.0 (0.2-10.6) | 0.368 |
| Baseline IL-8 | CTB fecal IgA | 16 | 2.9 (0.4-35.7) | 1.5 (0.2-10.6) | 0.394 |
| Baseline IL-10 | CS17 serum IgA | 4 | 0.4 (0.1-2.5) | 1.0 (0.2-2.8) | 0.105 |
| Baseline IL-10 | CS17 serum IgG | 8 | 0.6 (0.1-2.5) | 0.8 (0.1-2.8) | 0.789 |
| Baseline IL-10 | CS17 ALS IgA | 32 | 0.5 (0.1-2.5) | 0.8 (0.1-2.8) | 0.349 |
| Baseline IL-10 | CS17 ALS IgA | 64 | 0.7 (0.1 - 2.8) | 0.7 (0.1 - 2.1) | 0.948 |
| Baseline IL-10 | CS17 fecal IgA | 16 | 0.9 (0.1 - 2.5) | 0.8 (0.1 - 2.8) | 0.831 |
| Baseline IL-10 | CS17 fecal IgA | 32 | 1.1 (0.1-2.8) | 0.5 (0.1-1.7) | 0.199 |
| Baseline IL-10 | CTB ALS IgA | 8 | 0.5 (0.1-2.1) | 1.1 (0.2-2.8) | 0.228 |
| Baseline IL-10 | CTB ALS IgA | 16 | 0.7 (0.1-2.8) | 0.7 (0.2-2.5) | 1 |
| Baseline IL-10 | CTB fecal IgA | 16 | 0.8 (0.1-2.8) | 0.6 (0.1-2.5) | 0.748 |
| Baseline IL-10 | CTB fecal IgA | 32 | 0.6 (0.1-2.8) | 1.5 (0.8-2.5) | 0.493 |
| Baseline IL-13 | CS17 serum IgA | 8 | 7.8 (3.2-20.6) | 16.2 (7.1-25.6) | 0.061 |
| Baseline IL-13 | CS17 serum IgG | 8 | 9.6 (3.2-21.2) | 11.2 (3.2-25.6) | 0.592 |
| Baseline IL-13 | CS17 ALS IgA | 32 | 6.8 (3.2-16.3) | 12.7 (3.2-25.6) | 0.141 |
| Baseline IL-13 | CS17 ALS IgA | 64 | 8.4 (3.2 - 25.6) | 11.6 (3.2 - 25.0) | 0.56 |
| Baseline IL-13 | CS17 fecal IgA | 16 | 8.7 (3.2 - 21.2) | 11.4 (3.2 - 25.6) | 0.669 |
| Baseline IL-13 | CS17 fecal IgA | 32 | 12.1 (3.2-25.6) | 8.0 (3.2-16.3) | 0.392 |
| Baseline IL-13 | CTB ALS IgA | 8 | 8.9 (3.2-25.0) | 12.7 (7.1-25.6) | 0.688 |
| Baseline IL-13 | CTB ALS IgA | 16 | 9.6 (3.2-25.6) | 12.2 (9.9-16.3) | 1 |
| Baseline IL-13 | CTB fecal IgA | 16 | 12.1 (3.2-25.6) | 8.8 (3.2-16.3) | 0.336 |
| Baseline IL-13 | CTB fecal IgA | 32 | 10.0 (3.2-25.6) | 10.9 (7.1-16.3) | 0.819 |
| Baseline IL-17A | CS17 serum IgA | 4 | 1.7 (1.2-2.4) | 3.2 (1.2-7.7) | 0.05 |
| Baseline IL-17A | CS17 serum IgA | 8 | 2.1 (1.2-7.7) | 3.4 (2.4-6.2) | 0.106 |
| Baseline IL-17A | CS17 serum IgG | 8 | 2.4 (1.2-7.7) | 2.5 (1.6-4.5) | 0.788 |
| Baseline IL-17A | CS17 ALS IgA | 32 | 1.8 (1.2-2.4) | 3.0 (1.2-7.7) | 0.121 |
| Baseline IL-17A | CS17 ALS IgA | 64 | 1.9 (1.2 - 2.4) | 3.0 (1.2 - 7.7) | 0.09 |
| Baseline IL-17A | CS17 fecal IgA | 16 | 1.9 (1.2 - 3.0) | 3.2 (1.6 - 7.7) | 0.334 |
| Baseline IL-17A | CS17 fecal IgA | 32 | 2.5 (1.2-7.7) | 2.8 (1.6-6.2) | 0.748 |
| Baseline IL-17A | CTB ALS IgA | 8 | 2.6 (1.6-7.7) | 2.2 (1.2-6.2) | 0.346 |
| Baseline IL-17A | CTB ALS IgA | 16 | 2.8 (1.6-7.7) | 1.5 (1.2-2.4) | 0.084 |
| Baseline IL-17A | CTB fecal IgA | 16 | 2.8 (1.6-7.7) | 2.1 (1.2-6.2) | 0.257 |
| Baseline IL-17A | CTB fecal IgA | 32 | 2.6 (1.6-7.7) | 2.6 (1.2-6.2) | 0.909 |
| Baseline TNF-α | CS17 serum IgA | 4 | 1.8 (1.1-3.9) | 2.4 (0.9-7.2) | 0.468 |
| Baseline TNF-α | CS17 serum IgG | 8 | 1.8 (0.9-5.8) | 2.8 (1.1-7.2) | 0.495 |
| Baseline TNF-α | CS17 ALS IgA | 32 | 2.0 (1.1-3.9) | 2.2 (0.9-7.2) | 0.838 |
| Baseline TNF-α | CS17 ALS IgA | 64 | 2.4 (1.1 - 7.2) | 1.9 (0.9 - 5.8) | 0.597 |
| Baseline TNF-α | CS17 fecal IgA | 16 | 3.1 (1.1 - 5.8) | 1.9 (1.1 - 7.2) | 0.444 |
| Baseline TNF-α | CS17 fecal IgA | 32 | 3.2 (1.1-7.2) | 1.4 (1.1-1.8) | 0.101 |
| Baseline TNF-α | CTB ALS IgA | 8 | 2.0 (1.1-5.8) | 2.4 (0.9-7.2) | 0.682 |
| Baseline TNF-α | CTB ALS IgA | 16 | 2.2 (1.1-7.2) | 1.8 (0.9-3.9) | 0.691 |
| Baseline TNF-α | CTB fecal IgA | 16 | 2.6 (1.1-7.2) | 1.6 (0.9-3.9) | 0.412 |
| Baseline TNF-α | CTB fecal IgA | 32 | 2.3 (1.1-7.2) | 2.3 (1.8-3.9) | 0.815 |
| Baseline IFN-γ | CS17 serum IgA | 4 | 11.7 (8.8-35.4) | 14.6 (4.1-40.3) | 0.359 |
| Baseline IFN-γ | CS17 serum IgG | 8 | 14.3 (4.1-40.3) | 11.7 (8.8-23.2) | 0.892 |
| Baseline IFN-γ | CS17 ALS IgA | 32 | 12.2 (8.8-35.4) | 14.0 (4.1-40.3) | 0.416 |
| Baseline IFN-γ | CS17 ALS IgA | 64 | 13.5 (8.8 - 35.4) | 13.2 (4.1 - 40.3) | 0.743 |
| Baseline IFN-γ | CS17 fecal IgA | 16 | 14.9 (8.8 - 35.4) | 13.8 (8.8 - 40.3) | 0.913 |
| Baseline IFN-γ | CS17 fecal IgA | 32 | 14.7 (8.8-35.4) | 13.6 (8.8-40.3) | 1 |
| Baseline IFN-γ | CTB ALS IgA | 8 | 11.9 (8.8-36.6) | 16.4 (4.1-40.3) | 0.685 |
| Baseline IFN-γ | CTB ALS IgA | 16 | 14.1 (8.8-40.3) | 10.8 (4.1-35.4) | 0.343 |
| Baseline IFN-γ | CTB fecal IgA | 16 | 11.9 (8.8-23.2) | 16.2 (4.1-40.3) | 0.57 |
| Baseline IFN-γ | CTB fecal IgA | 32 | 11.6 (8.8-23.2) | 23.3 (8.8-40.3) | 0.35 |
| Peak IL-1β | CS17 serum IgA | 4 | 188.7 (43.4-1563.4) | 851.2 (74.8-4288.6) | 0.142 |
| Peak IL-1β | CS17 serum IgA | 8 | 327.0 (43.4-4195.1) | 781.5 (74.8-4288.6) | 0.438 |
| Peak IL-1β | CS17 serum IgG | 8 | 402.6 (59.4-4288.6) | 537.5 (43.4-4195.1) | 0.797 |
| Peak IL-1β | CS17 ALS IgA | 32 | 123.6 (43.4-706.5) | 910.7 (74.8-4288.6) | 0.042 |
| Peak IL-1β | CS17 ALS IgA | 64 | 165.1 (43.4 - 706.5) | 941.2 (74.8 - 4288.6) | 0.059 |
| Peak IL-1β | CS17 fecal IgA | 16 | 110.8 (43.4 - 693.4) | 1130.3 (149.9 - 4288.6) | 0.019 |
| Peak IL-1β | CS17 fecal IgA | 32 | 158.4 (43.4-700.0) | 2111.4 (706.5-4288.6) | 0.01 |
| Peak IL-1β | CTB ALS IgA | 8 | 329.9 (43.4-4195.1) | 769.2 (84.4-4288.6) | 0.364 |
| Peak IL-1β | CTB ALS IgA | 16 | 446.0 (43.4-4288.6) | 447.6 (84.4-1503.3) | 1 |
| Peak IL-1β | CTB fecal IgA | 16 | 303.8 (43.4-4195.1) | 596.4 (74.8-4288.6) | 0.31 |
| Peak IL-1β | CTB fecal IgA | 32 | 383.9 (43.4-4195.1) | 634.8 (84.4-4288.6) | 0.517 |
| Peak IL-2 | CS17 serum IgA | 4 | 3.7 (1.3-20.5) | 5.2 (2.2-13.8) | 0.573 |
| Peak IL-2 | CS17 serum IgA | 8 | 4.6 (1.3-20.5) | 4.4 (2.2-11.1) | 1 |
| Peak IL-2 | CS17 serum IgG | 8 | 5.1 (1.5-20.5) | 3.6 (1.3-13.8) | 0.606 |
| Peak IL-2 | CS17 ALS IgA | 32 | 3.9 (1.3-20.5) | 4.8 (2.2-13.8) | 0.797 |
| Peak IL-2 | CS17 ALS IgA | 64 | 3.8 (1.3 - 20.5) | 5.1 (2.2 - 13.8) | 0.662 |
| Peak IL-2 | CS17 fecal IgA | 16 | 5.2 (1.5 - 20.5) | 6.2 (2.8 - 13.8) | 1 |
| Peak IL-2 | CS17 fecal IgA | 32 | 5.2 (1.5-20.5) | 6.8 (2.8-13.8) | 0.914 |
| Peak IL-2 | CTB ALS IgA | 8 | 5.0 (1.3-20.5) | 3.7 (1.5-11.1) | 0.518 |
| Peak IL-2 | CTB ALS IgA | 16 | 5.2 (1.3-20.5) | 2.7 (1.5-4.9) | 0.225 |
| Peak IL-2 | CTB fecal IgA | 16 | 7.6 (3.0-20.5) | 3.7 (1.5-11.1) | 0.065 |
| Peak IL-2 | CTB fecal IgA | 32 | 6.5 (2.8-20.5) | 4.3 (1.5-11.1) | 0.517 |
| Peak IL-4 | CS17 serum IgA | 4 | 0.6 (0.1-1.6) | 0.9 (0.2-2.2) | 0.573 |
| Peak IL-4 | CS17 serum IgA | 8 | 0.6 (0.1-1.6) | 1.3 (0.7-2.2) | 0.06 |
| Peak IL-4 | CS17 serum IgG | 8 | 1.0 (0.3-2.2) | 0.5 (0.1-1.6) | 0.438 |
| Peak IL-4 | CS17 ALS IgA | 32 | 0.7 (0.1-1.6) | 0.8 (0.2-2.2) | 1 |
| Peak IL-4 | CS17 ALS IgA | 64 | 0.8 (0.1 - 1.6) | 0.8 (0.2 - 2.2) | 0.852 |
| Peak IL-4 | CS17 fecal IgA | 16 | 1.0 (0.7 - 1.6) | 0.8 (0.2 - 1.7) | 0.914 |
| Peak IL-4 | CS17 fecal IgA | 32 | 1.0 (0.7-1.6) | 0.7 (0.2-1.7) | 0.914 |
| Peak IL-4 | CTB ALS IgA | 8 | 0.6 (0.1-2.2) | 1.1 (0.6-1.7) | 0.298 |
| Peak IL-4 | CTB ALS IgA | 16 | 0.7 (0.1-2.2) | 1.0 (0.6-1.5) | 1 |
| Peak IL-4 | CTB fecal IgA | 16 | 0.8 (0.2-1.6) | 1.0 (0.3-2.2) | 0.589 |
| Peak IL-4 | CTB fecal IgA | 32 | 0.7 (0.2-1.6) | 1.4 (1.0-1.7) | 0.183 |
| Peak IL-6 | CS17 serum IgA | 4 | 1.8 (0.4-6.3) | 3.7 (1.3-9.2) | 0.108 |
| Peak IL-6 | CS17 serum IgG | 8 | 2.9 (1.1-9.2) | 2.5 (0.4-5.4) | 0.898 |
| Peak IL-6 | CS17 ALS IgA | 32 | 1.6 (0.4-6.3) | 3.6 (1.3-9.2) | 0.112 |
| Peak IL-6 | CS17 ALS IgA | 64 | 2.0 (0.4 - 6.3) | 3.5 (1.3 - 9.2) | 0.282 |
| Peak IL-6 | CS17 fecal IgA | 16 | 3.1 (1.5 - 6.3) | 3.0 (1.1 - 9.2) | 1 |
| Peak IL-6 | CS17 fecal IgA | 32 | 3.0 (1.5-6.3) | 3.0 (1.1-9.2) | 1 |
| Peak IL-6 | CTB ALS IgA | 8 | 2.4 (0.4-7.3) | 3.4 (1.1-9.2) | 0.606 |
| Peak IL-6 | CTB ALS IgA | 16 | 2.9 (0.4-9.2) | 2.1 (1.1-6.3) | 0.456 |
| Peak IL-6 | CTB fecal IgA | 16 | 2.7 (1.5-5.4) | 3.5 (1.1-9.2) | 0.699 |
| Peak IL-6 | CTB fecal IgA | 32 | 2.7 (1.5-5.4) | 4.0 (1.1-9.2) | 0.517 |
| Peak IL-8 | CS17 serum IgA | 4 | 85.3 (17.7-1124.7) | 199.4 (20.0-7663.6) | 0.662 |
| Peak IL-8 | CS17 serum IgA | 8 | 189.7 (17.7-7663.6) | 78.7 (20.0-2155.1) | 0.438 |
| Peak IL-8 | CS17 serum IgG | 8 | 198.8 (23.5-2155.1) | 72.3 (17.7-7663.6) | 0.147 |
| Peak IL-8 | CS17 ALS IgA | 32 | 50.9 (17.7-350.5) | 241.7 (20.0-7663.6) | 0.298 |
| Peak IL-8 | CS17 ALS IgA | 64 | 43.6 (17.7 - 350.5) | 330.1 (20.5 - 7663.6) | 0.081 |
| Peak IL-8 | CS17 fecal IgA | 16 | 44.7 (17.7 - 145.6) | 484.3 (20.0 - 7663.6) | 0.114 |
| Peak IL-8 | CTB ALS IgA | 8 | 102.1 (17.7-7663.6) | 240.0 (20.0-2155.1) | 0.518 |
| Peak IL-8 | CTB ALS IgA | 16 | 116.2 (17.7-7663.6) | 264.4 (48.5-1087.4) | 0.456 |
| Peak IL-8 | CTB fecal IgA | 16 | 103.8 (17.7-7663.6) | 319.1 (23.5-2155.1) | 0.31 |
| Peak IL-8 | CTB fecal IgA | 32 | 145.9 (17.7-7663.6) | 332.2 (48.5-2155.1) | 0.517 |
| Peak IL-10 | CS17 serum IgA | 4 | 1.1 (0.1-2.9) | 2.2 (0.8-3.4) | 0.181 |
| Peak IL-10 | CS17 serum IgA | 8 | 1.3 (0.1-2.9) | 2.4 (1.9-3.4) | 0.298 |
| Peak IL-10 | CS17 serum IgG | 8 | 1.9 (0.8-3.4) | 1.3 (0.1-2.9) | 1 |
| Peak IL-10 | CS17 ALS IgA | 32 | 1.0 (0.1-2.9) | 2.1 (0.8-3.4) | 0.298 |
| Peak IL-10 | CS17 ALS IgA | 64 | 1.2 (0.1 - 2.9) | 2.1 (0.8 - 3.4) | 0.573 |
| Peak IL-10 | CS17 fecal IgA | 16 | 1.7 (0.8 - 2.9) | 2.6 (1.8 - 3.4) | 0.171 |
| Peak IL-10 | CS17 fecal IgA | 32 | 2.0 (0.8-2.9) | 2.5 (1.8-3.4) | 0.352 |
| Peak IL-10 | CTB ALS IgA | 8 | 1.4 (0.1-2.9) | 2.2 (0.8-3.4) | 0.19 |
| Peak IL-10 | CTB ALS IgA | 16 | 1.6 (0.1-3.4) | 1.7 (0.8-2.9) | 1 |
| Peak IL-10 | CTB fecal IgA | 16 | 2.0 (0.8-2.9) | 2.0 (0.8-3.4) | 1 |
| Peak IL-10 | CTB fecal IgA | 32 | 2.0 (0.8-2.9) | 2.8 (2.2-3.4) | 0.183 |
| Peak IL-13 | CS17 serum IgA | 8 | 10.7 (3.2-23.7) | 23.7 (11.7-35.3) | 0.012 |
| Peak IL-13 | CS17 serum IgG | 8 | 14.3 (3.2-35.3) | 14.0 (7.7-25.6) | 0.797 |
| Peak IL-13 | CS17 ALS IgA | 32 | 9.8 (3.2-23.7) | 17.4 (5.6-35.3) | 0.147 |
| Peak IL-13 | CS17 ALS IgA | 64 | 11.5 (3.2 - 25.6) | 16.6 (5.6 - 35.3) | 0.414 |
| Peak IL-13 | CS17 fecal IgA | 16 | 11.3 (3.2 - 28.1) | 16.4 (5.6 - 35.3) | 0.762 |
| Peak IL-13 | CS17 fecal IgA | 32 | 14.3 (3.2-28.1) | 13.9 (5.6-35.3) | 0.914 |
| Peak IL-13 | CTB ALS IgA | 8 | 11.1 (3.2-28.1) | 22.0 (14.7-35.3) | 0.083 |
| Peak IL-13 | CTB ALS IgA | 16 | 13.3 (3.2-35.3) | 17.9 (14.7-23.7) | 0.769 |
| Peak IL-13 | CTB fecal IgA | 16 | 12.7 (3.2-28.1) | 15.4 (5.6-35.3) | 0.818 |
| Peak IL-13 | CTB fecal IgA | 32 | 11.3 (3.2-28.1) | 23.9 (16.3-35.3) | 0.267 |
| Peak IL-17A | CS17 serum IgA | 4 | 4.7 (1.6-9.4) | 8.8 (3.7-42.5) | 0.272 |
| Peak IL-17A | CS17 serum IgA | 8 | 5.5 (1.6-15.9) | 9.5 (4.5-42.5) | 0.593 |
| Peak IL-17A | CS17 serum IgG | 8 | 7.3 (1.6-42.5) | 5.8 (1.6-15.9) | 0.841 |
| Peak IL-17A | CS17 ALS IgA | 32 | 4.2 (1.6-9.4) | 8.7 (3.7-42.5) | 0.286 |
| Peak IL-17A | CS17 ALS IgA | 64 | 4.5 (1.6 - 9.4) | 9.0 (3.7 - 42.5) | 0.175 |
| Peak IL-17A | CS17 fecal IgA | 16 | 5.4 (1.6 - 9.4) | 11.3 (6.3 - 42.5) | 0.476 |
| Peak IL-17A | CS17 fecal IgA | 32 | 6.0 (1.6-9.4) | 14.2 (7.8-42.5) | 0.257 |
| Peak IL-17A | CTB ALS IgA | 8 | 5.7 (1.6-15.9) | 8.9 (3.7-42.5) | 0.894 |
| Peak IL-17A | CTB ALS IgA | 16 | 6.9 (1.6-42.5) | 6.0 (3.7-7.8) | 0.436 |
| Peak IL-17A | CTB fecal IgA | 16 | 6.8 (1.6-15.9) | 9.1 (3.7-42.5) | 0.699 |
| Peak IL-17A | CTB fecal IgA | 32 | 6.9 (1.6-15.9) | 13.5 (7.4-42.5) | 0.833 |
| Peak TNF-α | CS17 serum IgA | 4 | 5.5 (1.1-19.4) | 6.0 (2.8-15.0) | 1 |
| Peak TNF-α | CS17 serum IgA | 8 | 5.6 (1.1-19.4) | 6.1 (3.2-10.7) | 0.797 |
| Peak TNF-α | CS17 serum IgG | 8 | 6.4 (2.8-19.4) | 4.7 (1.1-15.0) | 0.699 |
| Peak TNF-α | CS17 ALS IgA | 32 | 4.2 (1.1-7.9) | 6.8 (2.8-19.4) | 0.438 |
| Peak TNF-α | CS17 ALS IgA | 64 | 4.6 (1.1 - 7.9) | 6.8 (2.8 - 19.4) | 0.662 |
| Peak TNF-α | CS17 fecal IgA | 16 | 6.0 (4.0 - 7.9) | 9.3 (4.7 - 19.4) | 0.257 |
| Peak TNF-α | CS17 fecal IgA | 32 | 5.9 (4.0-7.9) | 11.9 (6.4-19.4) | 0.067 |
| Peak TNF-α | CTB ALS IgA | 8 | 5.4 (1.1-19.4) | 6.4 (2.8-10.7) | 0.518 |
| Peak TNF-α | CTB ALS IgA | 16 | 5.9 (1.1-19.4) | 5.2 (2.8-7.9) | 0.885 |
| Peak TNF-α | CTB fecal IgA | 16 | 6.6 (4.0-15.0) | 6.8 (2.8-19.4) | 0.937 |
| Peak TNF-α | CTB fecal IgA | 32 | 7.7 (4.0-19.4) | 8.2 (6.4-10.7) | 0.667 |
| Peak IFN-γ | CS17 serum IgA | 4 | 25.0 (9.8-43.0) | 32.1 (10.9-138.6) | 0.561 |
| Peak IFN-γ | CS17 serum IgA | 8 | 25.1 (9.8-43.3) | 37.1 (10.9-138.6) | 0.423 |
| Peak IFN-γ | CS17 serum IgG | 8 | 37.2 (9.8-138.6) | 18.3 (9.8-43.0) | 0.125 |
| Peak IFN-γ | CS17 ALS IgA | 32 | 23.2 (9.8-43.0) | 32.5 (10.9-138.6) | 0.593 |
| Peak IFN-γ | CS17 ALS IgA | 64 | 23.8 (9.8 - 43.0) | 33.3 (10.9 - 138.6) | 0.477 |
| Peak IFN-γ | CS17 fecal IgA | 16 | 29.6 (9.8 - 47.0) | 40.0 (16.5 - 138.6) | 1 |
| Peak IFN-γ | CS17 fecal IgA | 32 | 31.1 (9.8-47.0) | 43.2 (16.5-138.6) | 1 |
| Peak IFN-γ | CTB ALS IgA | 8 | 23.3 (9.8-47.0) | 42.4 (22.5-138.6) | 0.423 |
| Peak IFN-γ | CTB ALS IgA | 16 | 27.8 (9.8-138.6) | 33.2 (22.5-41.9) | 0.876 |
| Peak IFN-γ | CTB fecal IgA | 16 | 27.0 (9.8-47.0) | 43.5 (22.5-138.6) | 0.818 |
| Peak IFN-γ | CTB fecal IgA | 32 | 28.1 (9.8-47.0) | 60.9 (38.8-138.6) | 0.383 |
| Peak IL-1β fold change | CS17 serum IgA | 4 | 80.1 (4.5-399.0) | 893.4 (31.1-15258.9) | 0.081 |
| Peak IL-1β fold change | CS17 serum IgG | 8 | 365.2 (31.1-15258.9) | 247.2 (4.5-10508.9) | 1 |
| Peak IL-1β fold change | CS17 ALS IgA | 32 | 58.1 (4.5-357.8) | 816.8 (31.1-15258.9) | 0.042 |
| Peak IL-1β fold change | CS17 ALS IgA | 64 | 84.9 (4.5 - 567.2) | 854.9 (31.1 - 15258.9) | 0.108 |
| Peak IL-1β fold change | CS17 fecal IgA | 16 | 109.6 (4.5 - 745.7) | 700.1 (31.1 - 15258.9) | 0.352 |
| Peak IL-1β fold change | CS17 fecal IgA | 32 | 116.9 (4.5-745.7) | 1607.4 (329.2-15258.9) | 0.171 |
| Peak IL-1β fold change | CTB ALS IgA | 8 | 198.7 (4.5-10508.9) | 739.5 (216.9-15258.9) | 0.438 |
| Peak IL-1β fold change | CTB ALS IgA | 16 | 324.4 (4.5-15258.9) | 294.5 (216.9-357.8) | 0.769 |
| Peak IL-1β fold change | CTB fecal IgA | 16 | 169.5 (4.5-3331.1) | 588.2 (216.9-15258.9) | 0.699 |
| Peak IL-1β fold change | CTB fecal IgA | 32 | 191.6 (4.5-3331.1) | 1215.8 (329.2-15258.9) | 0.667 |
| Peak IL-2 fold change | CS17 serum IgA | 4 | 3.9 (1.0-35.3) | 4.8 (1.0-23.9) | 0.438 |
| Peak IL-2 fold change | CS17 serum IgA | 8 | 5.1 (1.0-35.3) | 3.5 (1.0-7.7) | 0.841 |
| Peak IL-2 fold change | CS17 serum IgG | 8 | 5.2 (2.3-35.3) | 3.3 (1.0-23.9) | 0.423 |
| Peak IL-2 fold change | CS17 ALS IgA | 32 | 3.8 (1.0-35.3) | 4.8 (1.0-23.9) | 0.257 |
| Peak IL-2 fold change | CS17 ALS IgA | 64 | 3.9 (1 - 35.3) | 4.9 (1 - 23.9) | 0.366 |
| Peak IL-2 fold change | CS17 fecal IgA | 16 | 6.0 (2.3 - 35.3) | 6.4 (2.7 - 23.9) | 0.476 |
| Peak IL-2 fold change | CS17 fecal IgA | 32 | 5.8 (2.3-35.3) | 7.0 (2.7-23.9) | 0.61 |
| Peak IL-2 fold change | CTB ALS IgA | 8 | 4.7 (1.0-35.3) | 3.9 (2.3-7.7) | 0.789 |
| Peak IL-2 fold change | CTB ALS IgA | 16 | 4.9 (1.0-35.3) | 2.9 (2.3-4.0) | 0.212 |
| Peak IL-2 fold change | CTB fecal IgA | 16 | 8.5 (3.4-35.3) | 3.8 (2.3-7.7) | 0.132 |
| Peak IL-2 fold change | CTB fecal IgA | 32 | 7.8 (3.4-35.3) | 3.6 (2.3-7.7) | 0.267 |
| Peak IL-4 fold change | CS17 serum IgA | 4 | 3.3 (1.4-9.4) | 1.9 (1.0-5.2) | 0.137 |
| Peak IL-4 fold change | CS17 serum IgA | 8 | 2.6 (1.0-9.4) | 2.1 (1.0-5.2) | 0.64 |
| Peak IL-4 fold change | CS17 serum IgG | 8 | 2.7 (1.0-9.4) | 2.0 (1.0-3.3) | 0.64 |
| Peak IL-4 fold change | CS17 ALS IgA | 32 | 3.2 (1.4-9.4) | 2.1 (1.0-5.2) | 0.286 |
| Peak IL-4 fold change | CS17 ALS IgA | 64 | 2.9 (1.4 - 9.4) | 2.1 (1 - 5.2) | 0.401 |
| Peak IL-4 fold change | CS17 fecal IgA | 16 | 2.7 (1.4 - 9.4) | 2.7 (1.4 - 4.3) | 0.914 |
| Peak IL-4 fold change | CS17 fecal IgA | 32 | 2.3 (1.4-9.4) | 3.6 (2.7-4.3) | 0.171 |
| Peak IL-4 fold change | CTB ALS IgA | 8 | 2.7 (1.0-9.4) | 2.0 (1.0-4.3) | 0.463 |
| Peak IL-4 fold change | CTB ALS IgA | 16 | 2.6 (1.0-9.4) | 1.8 (1.0-4.3) | 0.483 |
| Peak IL-4 fold change | CTB fecal IgA | 16 | 2.6 (1.4-9.4) | 2.7 (1.0-5.2) | 0.937 |
| Peak IL-4 fold change | CTB fecal IgA | 32 | 2.8 (1.4-9.4) | 2.6 (1.4-4.3) | 1 |
| Peak IL-6 fold change | CS17 serum IgA | 4 | 2.8 (1.0-7.4) | 3.6 (1.0-18.4) | 0.796 |
| Peak IL-6 fold change | CS17 serum IgA | 8 | 3.0 (1.0-7.5) | 3.6 (1.0-18.4) | 1 |
| Peak IL-6 fold change | CS17 serum IgG | 8 | 4.0 (1.2-18.4) | 2.2 (1.0-7.5) | 0.23 |
| Peak IL-6 fold change | CS17 ALS IgA | 32 | 2.3 (1.0-5.7) | 3.9 (1.0-18.4) | 0.463 |
| Peak IL-6 fold change | CS17 ALS IgA | 64 | 2.0 (1 - 5.7) | 4.5 (1 - 18.4) | 0.196 |
| Peak IL-6 fold change | CS17 fecal IgA | 16 | 2.7 (1.2 - 5.7) | 4.5 (1.1 - 18.4) | 0.476 |
| Peak IL-6 fold change | CS17 fecal IgA | 32 | 2.4 (1.1-5.7) | 6.9 (2.2-18.4) | 0.067 |
| Peak IL-6 fold change | CTB ALS IgA | 8 | 3.6 (1.0-14.5) | 2.5 (1.1-18.4) | 0.593 |
| Peak IL-6 fold change | CTB ALS IgA | 16 | 3.8 (1.0-18.4) | 1.7 (1.2-2.2) | 0.35 |
| Peak IL-6 fold change | CTB fecal IgA | 16 | 3.3 (1.1-7.5) | 4.6 (1.2-18.4) | 0.699 |
| Peak IL-6 fold change | CTB fecal IgA | 32 | 3.7 (1.1-7.5) | 3.6 (1.2-18.4) | 1 |
| Peak IL-8 fold change | CS17 serum IgA | 4 | 69.7 (3.7-801.0) | 54.7 (2.8-5397.6) | 0.95 |
| Peak IL-8 fold change | CS17 serum IgA | 8 | 82.5 (2.8-5397.6) | 35.0 (6.0-1657.5) | 0.797 |
| Peak IL-8 fold change | CS17 serum IgG | 8 | 100.3 (2.8-1657.5) | 24.6 (3.7-5397.6) | 0.364 |
| Peak IL-8 fold change | CS17 ALS IgA | 32 | 45.7 (3.7-801.0) | 71.0 (2.8-5397.6) | 0.797 |
| Peak IL-8 fold change | CS17 ALS IgA | 64 | 34.8 (3.7 - 801.0) | 92.0 (2.8 - 5397.6) | 0.573 |
| Peak IL-8 fold change | CS17 fecal IgA | 16 | 41.4 (3.7 - 205.5) | 216.3 (2.8 - 5397.6) | 0.352 |
| Peak IL-8 fold change | CTB ALS IgA | 8 | 32.2 (2.8-5397.6) | 190.3 (9.0-1657.5) | 0.147 |
| Peak IL-8 fold change | CTB ALS IgA | 16 | 41.0 (2.8-5397.6) | 256.2 (102.1-801.0) | 0.225 |
| Peak IL-8 fold change | CTB fecal IgA | 16 | 35.3 (2.8-5397.6) | 213.7 (5.9-1657.5) | 0.18 |
| Peak IL-8 fold change | CTB fecal IgA | 32 | 52.6 (2.8-5397.6) | 648.6 (205.5-1657.5) | 0.183 |
| Peak IL-10 fold change | CS17 serum IgA | 4 | 2.9 (1.0-14.3) | 2.1 (1.0-8.4) | 0.56 |
| Peak IL-10 fold change | CS17 serum IgG | 8 | 2.9 (1.1-14.3) | 1.8 (1.0-8.4) | 0.141 |
| Peak IL-10 fold change | CS17 ALS IgA | 32 | 2.1 (1.0-6.6) | 2.6 (1.0-14.3) | 0.893 |
| Peak IL-10 fold change | CS17 ALS IgA | 64 | 1.9 (1 - 6.6) | 3.0 (1 - 14.3) | 0.399 |
| Peak IL-10 fold change | CS17 fecal IgA | 16 | 2.0 (1.1 - 6.6) | 3.5 (1.0 - 14.3) | 0.352 |
| Peak IL-10 fold change | CS17 fecal IgA | 32 | 1.8 (1.0-6.6) | 5.2 (2.1-14.3) | 0.067 |
| Peak IL-10 fold change | CTB ALS IgA | 8 | 2.7 (1.0-14.3) | 2.0 (1.0-4.5) | 0.893 |
| Peak IL-10 fold change | CTB ALS IgA | 16 | 2.4 (1.0-14.3) | 2.4 (1.1-4.5) | 0.639 |
| Peak IL-10 fold change | CTB fecal IgA | 16 | 2.6 (1.0-8.4) | 3.1 (1.1-14.3) | 0.699 |
| Peak IL-10 fold change | CTB fecal IgA | 32 | 3.3 (1.0-14.3) | 1.9 (1.1-2.8) | 0.833 |
| Peak IL-13 fold change | CS17 serum IgA | 4 | 1.5 (1.0-3.1) | 1.3 (1.0-5.0) | 0.567 |
| Peak IL-13 fold change | CS17 serum IgA | 8 | 1.4 (1.0-3.1) | 1.5 (1.0-5.0) | 0.941 |
| Peak IL-13 fold change | CS17 serum IgG | 8 | 1.5 (1.0-5.0) | 1.3 (1.0-3.1) | 0.376 |
| Peak IL-13 fold change | CS17 ALS IgA | 32 | 1.5 (1.0-3.1) | 1.4 (1.0-5.0) | 0.941 |
| Peak IL-13 fold change | CS17 ALS IgA | 64 | 1.4 (1 - 3.1) | 1.4 (1 - 5.0) | 0.83 |
| Peak IL-13 fold change | CS17 fecal IgA | 16 | 1.3 (1.0 - 2.1) | 1.4 (1.0 - 5.0) | 0.904 |
| Peak IL-13 fold change | CS17 fecal IgA | 32 | 1.2 (1.0-2.1) | 1.7 (1.0-5.0) | 0.548 |
| Peak IL-13 fold change | CTB ALS IgA | 8 | 1.2 (1.0-3.1) | 1.7 (1.0-5.0) | 0.301 |
| Peak IL-13 fold change | CTB ALS IgA | 16 | 1.4 (1.0-5.0) | 1.5 (1.0-2.1) | 0.546 |
| Peak IL-13 fold change | CTB fecal IgA | 16 | 1.0 (1.0-1.3) | 1.7 (1.0-5.0) | 0.061 |
| Peak IL-13 fold change | CTB fecal IgA | 32 | 1.1 (1.0-1.8) | 2.2 (1.0-5.0) | 0.158 |
| Peak IL-17A fold change | CS17 serum IgA | 4 | 2.7 (1.0-6.0) | 2.7 (1.0-6.8) | 1 |
| Peak IL-17A fold change | CS17 serum IgA | 8 | 2.7 (1.0-6.4) | 2.7 (1.0-6.8) | 0.893 |
| Peak IL-17A fold change | CS17 serum IgG | 8 | 3.0 (1.0-6.8) | 2.3 (1.0-6.4) | 0.503 |
| Peak IL-17A fold change | CS17 ALS IgA | 32 | 2.4 (1.0-6.0) | 2.9 (1.0-6.8) | 0.688 |
| Peak IL-17A fold change | CS17 ALS IgA | 64 | 2.4 (1 - 6.0) | 3.0 (1 - 6.8) | 0.476 |
| Peak IL-17A fold change | CS17 fecal IgA | 16 | 2.8 (1.0 - 6.0) | 3.5 (1.0 - 6.8) | 0.61 |
| Peak IL-17A fold change | CS17 fecal IgA | 32 | 2.4 (1.0-6.0) | 5.1 (3.2-6.8) | 0.067 |
| Peak IL-17A fold change | CTB ALS IgA | 8 | 2.2 (1.0-6.4) | 4.0 (2.6-6.8) | 0.284 |
| Peak IL-17A fold change | CTB ALS IgA | 16 | 2.5 (1.0-6.8) | 3.9 (3.0-6.0) | 0.532 |
| Peak IL-17A fold change | CTB fecal IgA | 16 | 2.4 (1.0-6.4) | 4.3 (3.0-6.8) | 0.132 |
| Peak IL-17A fold change | CTB fecal IgA | 32 | 2.6 (1.0-6.4) | 5.1 (3.2-6.8) | 0.183 |
| Peak TNF-α fold change | CS17 serum IgA | 4 | 3.1 (1.0-18.3) | 2.5 (1.0-14.1) | 0.746 |
| Peak TNF-α fold change | CS17 serum IgA | 8 | 3.6 (1.0-18.3) | 1.7 (1.0-6.1) | 0.18 |
| Peak TNF-α fold change | CS17 serum IgG | 8 | 3.5 (1.1-18.3) | 1.7 (1.0-14.1) | 0.061 |
| Peak TNF-α fold change | CS17 ALS IgA | 32 | 2.2 (1.0-5.9) | 3.1 (1.0-18.3) | 0.688 |
| Peak TNF-α fold change | CS17 ALS IgA | 64 | 1.9 (1 - 5.9) | 3.6 (1 - 18.3) | 0.27 |
| Peak TNF-α fold change | CS17 fecal IgA | 16 | 1.9 (1.1 - 5.9) | 5.0 (1.0 - 18.3) | 0.257 |
| Peak TNF-α fold change | CTB ALS IgA | 8 | 2.7 (1.0-18.3) | 2.7 (1.0-6.1) | 0.789 |
| Peak TNF-α fold change | CTB ALS IgA | 16 | 2.7 (1.0-18.3) | 2.9 (2.0-3.6) | 0.639 |
| Peak TNF-α fold change | CTB fecal IgA | 16 | 2.5 (1.0-14.1) | 4.1 (1.8-18.3) | 0.31 |
| Peak TNF-α fold change | CTB fecal IgA | 32 | 3.4 (1.0-18.3) | 3.6 (2.0-6.1) | 0.833 |
| Peak IFN-γ fold change | CS17 serum IgA | 4 | 2.1 (1.0-4.9) | 2.2 (1.0-5.6) | 0.845 |
| Peak IFN-γ fold change | CS17 serum IgA | 8 | 2.5 (1.0-5.6) | 1.6 (1.0-3.4) | 0.225 |
| Peak IFN-γ fold change | CS17 serum IgG | 8 | 2.6 (1.0-5.6) | 1.6 (1.0-4.9) | 0.345 |
| Peak IFN-γ fold change | CS17 ALS IgA | 32 | 1.9 (1.0-4.9) | 2.3 (1.0-5.6) | 0.59 |
| Peak IFN-γ fold change | CS17 ALS IgA | 64 | 1.8 (1 - 4.9) | 2.5 (1 - 5.6) | 0.473 |
| Peak IFN-γ fold change | CS17 fecal IgA | 16 | 2.0 (1.0 - 4.9) | 2.9 (1.2 - 4.9) | 0.352 |
| Peak IFN-γ fold change | CS17 fecal IgA | 32 | 2.1 (1.0-4.9) | 3.2 (1.7-4.8) | 0.61 |
| Peak IFN-γ fold change | CTB ALS IgA | 8 | 2.0 (1.0-4.9) | 2.6 (1.1-5.6) | 0.345 |
| Peak IFN-γ fold change | CTB ALS IgA | 16 | 2.0 (1.0-4.9) | 3.1 (1.1-5.6) | 0.27 |
| Peak IFN-γ fold change | CTB fecal IgA | 16 | 2.3 (1.0-4.9) | 2.7 (1.0-5.6) | 0.873 |
| Peak IFN-γ fold change | CTB fecal IgA | 32 | 2.4 (1.0-4.9) | 2.6 (1.1-4.8) | 1 |
| Data were displayed as geometric mean (range). Cytokine concentrations were shown as ng/g. Antibody fold change cut off: cut off of antibody titers fold change from baseline to peak. | | | | | |
